# Plateau depolarizations in spontaneously active neurons detected by calcium or voltage imaging

**DOI:** 10.1038/s41598-024-70319-4

**Published:** 2024-10-04

**Authors:** Katarina D. Milicevic, Violetta O. Ivanova, Darko D. Lovic, Jelena Platisa, Pavle R. Andjus, Srdjan D. Antic

**Affiliations:** 1 School of Medicine, Institute for Systems Genomics, UConn Health, University of Connecticut Health, 263 Farmington Avenue, Farmington, CT 06030 USA; 2 Institute of Physiology and Biochemistry ‘Jean Giaja’, Center for Laser Microscopy, University of Belgrade, Faculty of Biology, 11000 Belgrade, Serbia; 3 The John B. Pierce Laboratory, New Haven, CT 06519 USA; 4 Department of Cellular and Molecular Physiology, School of Medicine, Yale University, New Haven, CT 06519 USA

**Keywords:** Biological techniques, Biophysics, Neuroscience, Physiology

## Abstract

In calcium imaging studies, Ca^2+^ transients are commonly interpreted as neuronal action potentials (APs). However, our findings demonstrate that robust optical Ca^2+^ transients primarily stem from complex “AP-Plateaus”, while simple APs lacking underlying depolarization envelopes produce much weaker photonic signatures. Under challenging in vivo conditions, these “AP-Plateaus” are likely to surpass noise levels, thus dominating the Ca^2+^ recordings. In spontaneously active neuronal culture, optical Ca^2+^ transients (OGB1-AM, GCaMP6f) exhibited approximately tenfold greater amplitude and twofold longer half-width compared to optical voltage transients (ArcLightD). The amplitude of the ArcLightD signal exhibited a strong correlation with the duration of the underlying membrane depolarization, and a weaker correlation with the presence of a fast sodium AP. Specifically, ArcLightD exhibited robust responsiveness to the slow “foot” but not the fast “trunk” of the neuronal AP. Particularly potent stimulators of optical signals in both Ca^2+^ and voltage imaging modalities were APs combined with plateau potentials (AP-Plateaus), resembling dendritic Ca^2+^ spikes or “UP states” in pyramidal neurons. Interestingly, even the spikeless plateaus (amplitude > 10 mV, duration > 200 ms) could generate conspicuous Ca^2+^ optical signals in neurons. Therefore, in certain circumstances, Ca^2+^ transients should not be interpreted solely as indicators of neuronal AP firing.

## Introduction

Recording electrical activity from multiple neurons simultaneously improves the robustness of results and decreases the number of experimental sweeps needed to detect significant difference between experimental conditions, e.g., before and after application of a drug^1^. Due to its substantial optical signals, *Ca*^*2*+^ *imaging* has long been considered well-suited for this purpose and has been successfully used to study spontaneous neuronal activity^1,2^. However, *voltage imaging* has recently emerged with several potential advantages not achievable through *Ca*^*2*+^ *imaging*: (1) direct assessment of membrane potential changes; (2) precise quantification of the rising and falling phases of neuronal electrical signaling; (3) accurate counting of neuronal spikes; and (4) detection of subthreshold depolarizations, which are believed to significantly contribute to synaptic integration^3,4^ and network synchronicity^5–7^.

The intrinsic disparities in kinetics between calcium and voltage signals impose constraints on the imaging speeds required for accurate signal detection. For instance, in *Ca*^*2*+^ *imaging* mode, it is feasible to sample optical signals at a video frame rate of 30 Hz or lower, without sacrificing vital information about ongoing neuronal activity^8,9^. Conversely, in *voltage imaging* mode, the video frame rate must be at least tenfold faster (300 Hz)^10^ or even 30-fold faster (up to 1000 Hz)^11–16^, to retain crucial details of neuronal electrical signaling, such as spike count and the temporal dynamics of signal rise and decay^17^. While some researchers successfully capture GEVIs at rates below 100 Hz, yielding valuable data^11,18^, conducting multi-cell optical imaging at faster rates poses technical challenges. Advanced technical solutions, such as rapid scanning^19–22^ or patterned illumination^23,24^, are necessary to address multiple regions of interest. Additionally, at higher sampling rates, fewer photons are accumulated per sampling point, leading to an increase in baseline optical noise. Moreover, when responding to a biological signal, Ca^2+^ indicators demonstrate significantly greater changes in resting fluorescence (F) compared to voltage indicators. Consequently, the signal-to-noise ratio (SNR) in *voltage imaging* experiments is typically tenfold smaller on average than in *Ca*^*2*+^ *imaging* experiments^25^. For all of the above, *voltage imaging* is technically more challenging than *Ca*^*2*+^ *imaging*^26,27^.

It has been suggested that optical signals from *Ca*^2+^ *imaging* and *voltage imaging* reflect neuronal spiking, specifically the generation of fast action potentials (APs)^9,28^. Our investigation revealed that the three optical indicators tested, OGB1-AM, GCaMP6f, and ArcLightD, exhibited the strongest responses to electrical signals that combine APs with plateau potentials (AP-Plateaus). Occasionally, optical traces from Ca^2+^ and voltage imaging contained transients that were not solely caused by sodium APs, but rather by spikeless 10–20 mV depolarizations lasting 200–500 ms (spikeless plateaus). In essence, optical recordings lacking simultaneous intracellular recordings are generally unable to discern the underlying electrical phenomena. Spikeless neuronal depolarizations (Plateaus), along with plateaus combined with APs (AP-Plateaus), constitute a significant portion of recorded optical transients and, therefore, should be considered when interpreting optical recordings and contextualizing them within correlated network activity^29^.

## Results

### Ca^2+^ imaging

**Figure 1:**
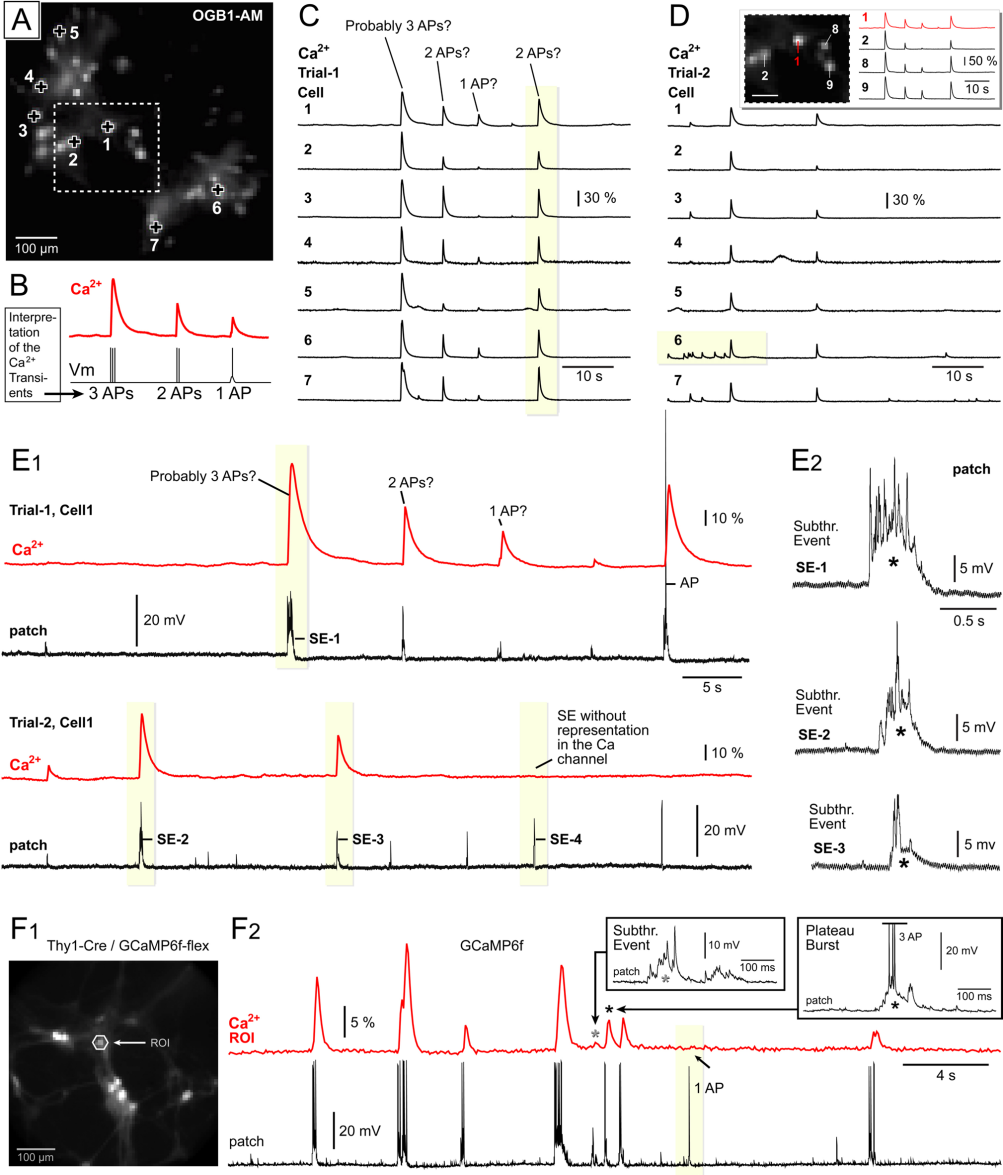
Interpretation of Ca^2+^ signals. (**A**) Cultured neurons are loaded with OGB1-AM and photographed at 14 Hz rate. (**B**) Relation between the Vm and Ca2 + . (**C**) Simultaneous Ca2 + imaging of spontaneous activity in seven neurons (Cells 1–7). Labels atop the Ca2 + trace indicate attempts to classify individual transients based on their most probable electrical underpinnings. A shaded vertical stripe marks a synchronized network event in all seven cells. The amplitude scale (30% ΔF/F) applies solely to Cell #3, with other cells arbitrarily scaled for display. (**D**) Similar to Panel *C*, showcasing a new experimental trial (Trial-2). A shaded horizontal stripe indicates a period during which Cell #6 exhibits independent activity. Inset: Ca2 + signals from the three nearest neurons (#2, #8, and #9) align with the activity of neuron #1. (**E**_**1**_) Dual electrical and optical recordings obtained in two consecutive experimental trials. Events labeled “3 APs”, “2 APs”, and “1 AP” in Panel *A* have been misinterpreted; instead, subthreshold events (SE) generate these Ca2 + transients. (**E**_**2**_) A sustained depolarization (asterisk) underlies each SE. (**F**_**1**_) Cultured neurons expressing GCaMP6f. (**F**_**2**_) Similar experimental setup as in Panel *E*_*1*_, except GCaMP6f is utilized to detect Ca2 + . Insets: Sustained depolarizations (asterisks) producing Ca2 + signals are displayed on a finer scale. A single action potential (1 AP) lacking the underlying sustained depolarization (plateau) is not observed in the Ca2 + imaging channel (arrow).

Depolarization of the neuronal membrane produces an influx of Ca^2+^ ions, which can be detected by optical measurements^30^. This feature has been exploited in many recent studies to monitor activity in many neurons simultaneously at low optical sampling speeds, e.g. 3–30 Hz^31–33^. Using a 10 × objective lens and a 14 Hz sampling rate, we conducted simultaneous recordings of Ca^2+^ transients from 10 to 40 neurons in culture (Fig. 1A). With low illumination intensity (less than 10% LED power, 1.4 mW/mm^2^), multiple optical sweeps can be obtained from the same visual field, a critical aspect for experimental designs exploring neuronal physiology before and after acute treatment with chemical or physical agents (e.g., electrolytes, drugs, temperature, radiation, etc.). Two successive recording trials, each lasting 72 s, are displayed in Fig. 1C and D. On average, we detected 10.7 ± 0.7 Ca^2+^ transients per recording trial (n = 78 trials, 12 coverslips), equivalent to 8.9 ± 1.05 Ca^2+^ events per minute. The occurrence of large-amplitude Ca^2+^ transients in multiple neurons simultaneously (Fig. 1C, vertical-yellow stripe) suggests strong synaptic connections between neurons and synchronized network activity. Neurons located at varying distances from each other are more likely to exhibit dissimilar firing patterns (Fig. 1D, comparing cells #1 and #6, horizontal-yellow stripe). Conversely, neurons in close proximity to each other tend to exhibit similar patterns of spontaneous activity (Fig. 1D, *Inset*, comparing neighboring Cells #1, #2, #8, and #9). These findings support the idea that cultured neurons are more inclined to extend their axons and establish synaptic contacts with neighboring cells compared to more distant ones.

#### The Ca^2+^-imaging method does not exclusively detect action potentials (APs)

Interpretation of brief Ca^2+^ transients in electrically-active neurons typically relies on two assumptions. Firstly, it is assumed that Ca^2+^ transients observed in the neuronal cell body (soma) are caused by action potentials (APs)^9,34, 35^. Secondly, it is assumed that the optical Ca^2+^ signal amplitude is roughly proportional to the number of consecutive spikes (Fig. 1B)^9,34, 35^. Initially, we classified Ca^2+^ transients solely based on optical signal amplitude, where a larger amplitude indicated a higher number of underlying APs (Fig. 1B). Labels above traces (Fig. 1C, *Probably 3APs?*) represent our educated guesses—a standard procedure for interpreting a Ca^2+^ imaging trace in the absence of direct electrical recording^34,36^. However, Cell #1 was patched, and the electrical recording was synchronized with the Ca^2+^ optical trace (Fig. 1E_1_). We found that some of the Ca^2+^ transients were not initiated by APs, but rather by subthreshold events (SEs) with amplitudes not exceeding 20 mV (Fig. 1E_1_). A slow component of depolarization (Fig. 1E_2_, asterisk) played a crucial role in triggering the Ca^2+^ signal. To eliminate a possibility that a calcium-sensitive dye (OGB1-AM) was somehow responsible for the observed relationship between optical and electrical signaling, we conducted a series of experiments (n = 4) using a genetically-encoded Ca^2+^ indicator (GCaMP6f) expressed in pyramidal neurons (Fig. 1F_1_; Suppl. Fig. S1-A). Dual electrical-optical recordings in GCaMP6f-expressing neurons confirmed that the strongest Ca^2+^ transients were associated with subthreshold depolarizations (Fig. 1F_2_, asterisk; Suppl. Fig. S1-C). In summary, a significant number of strong Ca^2+^ signals in both OGB1-AM (Fig. 1E_2_ and GCaMP6f optical recordings (Fig. 1F_2_) did not originate from “simple” action potentials (APs), but rather from electrical events combining APs with sustained depolarizations (plateaus).

#### Ca^2+^ signals from neighboring neurons can contaminate ROI

Similar to in vivo recordings, in neuronal cultures as well, the cell bodies, dendrites, and axons of neighboring neurons often overlap, whether we use neuron bulk-loading with OGB1-AM (Fig. 1A) or GCaMP6f expression (Fig. 1F_1_). Consequently, two or more cells may contribute calcium transients to the same region of interest (ROI), complicating the interpretation of multi-cell calcium imaging data (Fig. 1F_2_), and necessitating various demixing strategies^37^.

#### One-cell Ca^2+^ imaging—Simple APs induce small optical signals

**Figure 2:**
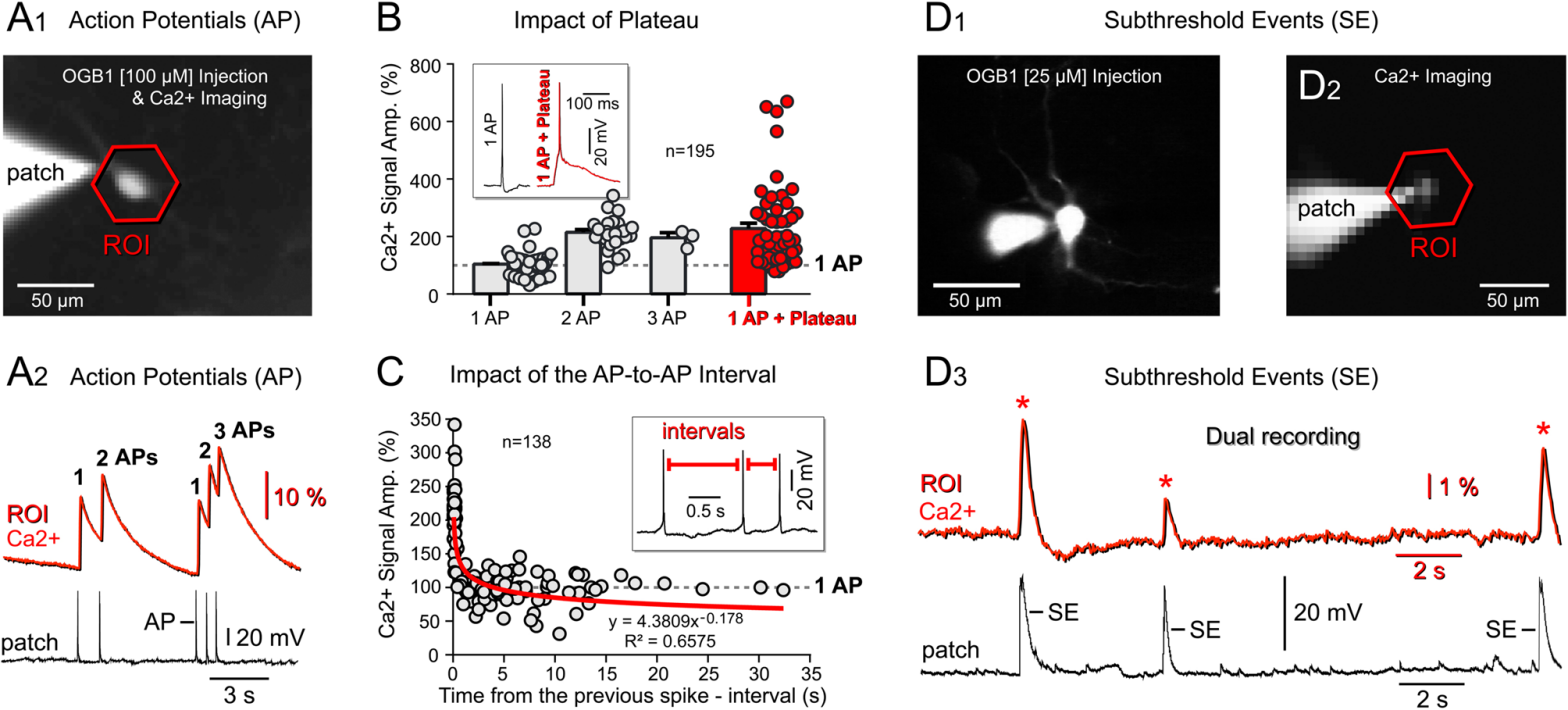
Subthreshold events produce Ca^2+^ optical signals. (**A**_**1**_) Intracellular injection of a Ca^2+^-sensitive dye, OGB1 [100 μM] followed by Ca^2+^ imaging. (**A**_**2**_) Dual electrical (patch) and optical (Ca^2+^) recording of subsequent APs. (**B**) Scatter plot depicting normalized Ca^2+^ signal amplitude versus type of electrical event. A horizontal dashed gray line indicates the trace-specific average amplitude of Ca^2+^ signals caused by confirmed single action potential (1 AP) events. Each data point represents one electrical event measured both electrically (via patch) and optically (via Ca^2+^ imaging). The scatter plot comprises 195 events obtained from 9 neurons via 30 dual-recording traces (each trace lasting 30 s). Inset: Whole-cell recordings of “*1 AP*” and “*1 AP with plateau*”. (**C**) Scatter plot illustrating normalized Ca^2+^ signal amplitude versus the time elapsed since the previous spike (AP-to-AP interval). This dataset excludes plateau events (n = 57). Inset: Red bars delineate intervals between electrically-recorded action potentials (*AP-to-AP interval*). (**D**_**1**_) Neuron injected with OGB1 [25 μM]. (**D**_**2**_) Optical imaging of neuronal spontaneous activity using low illumination intensity (neutral density filter, 0.05) and a camera frame rate of 28 Hz (sampling interval = 36 ms). (**D**_**3**_) Red optical trace (obtained by averaging 11 pixels within the region of interest, ROI) and whole-cell recording (black trace) are time-aligned. Spontaneous subthreshold events (SE) trigger noticeable optical signals.

To eliminate contamination from neighboring cells experimentally, rather than relying solely on computer algorithms^37^, we loaded individual neurons with a membrane-impermeable Ca^2+^-sensitive dye, OGB1 [100 μM], via a patch pipette. To ensure the accuracy of our approach, we used the fluorescent channel to confirm that only one cell was loaded with the dye (Fig. 2A_1_). Subsequently, neurons (n = 8) were allowed to generate spontaneous activity. In our “single-cell” experiments, two out of 8 neurons exhibited a good correlation between Ca^2+^ and whole-cell channels (Fig. 2A_1,2_). This correlation was evident as each individual action potential (AP) was distinctly represented in the Ca^2+^-imaging channel either as a clear peak, or as a proportional increase in optical signal amplitude corresponding to the number of underlying APs detected in the whole-cell channel (Suppl. Fig. S2). In 6 out of the 8 cells, a complex electrical waveform, characterized by an AP riding atop a plateau depolarization (AP-Plateau), triggered robust calcium transients (Suppl. Fig. S3, “AP-Plateau”). These *AP-Plateaus* not only generated strong somatic Ca^2+^ signals but also propagated into the dendrites of cultured cortical neurons to produce dendritic Ca^2+^ transients (Suppl. Fig. S4, events “*p”* and “*t”*). Conversely, single APs lacking the sustained depolarization at their base, often exhibited weak representations in the Ca^2+^-imaging channel (Suppl. Fig. S3, events #2, 5 & 6), akin to in vivo dual voltage-calcium recordings^11^, their figure five. Specifically, in 8 cells (24 recording trials, trial duration 72 s), we identified 156 simple individual APs (lacking plateau depolarization). Out of 156 simple APs, 60 spikes (38.4%) failed to induce a Ca^2+^ optical signal that rose 3 standard deviations above the baseline (Suppl. Fig. S3, events #5 & 6). It is worth noting that a different Ca^2+^ indicator (e.g. Cal-520, OGB5N, etc.) may exhibit faster kinetics^38,39^, potentially reporting neuronal action potential firing more efficiently^40^. Next, all electrical events were quantified in the OGB1 Ca^2+^-trace, normalized by the average 1-AP amplitude for that optical trace, and plotted in Fig. 2B. Electrical events comprising 1-AP riding on top of plateau depolarization (inset) showed the greatest optical signals (Fig. 2B, red dots). A combination of 1-AP with plateau gave on average twofold larger Ca^2+^ transients compared to a simple 1-AP event (Fig. 2B, compare “1-AP” vs. “1-AP + Plateau”).

#### One-cell Ca^2+^ imaging—Plateaus boost optical signals

The residual intracellular Ca^2+^ concentration is expected to depend on the time interval between two subsequent spikes. We can observe this relationship only if we omit 57 data points labeled “*1-AP* + *Plateau*”. In the remaining ‘simple spike data points (n = 138)’, exclusively made of APs that lack plateaus, the Ca^2+^-signal amplitude was exponentially dependent on the amount of time between two consecutive APs (AP-to-AP interval, Fig. 2C). Time intervals shorter than 2 s triggered a supralinear jump in the optical signal amplitude due to the buildup of intracellular Ca^2+^. The dependance of the optical signal amplitude on the time interval is best described by the function displayed below the red tradeline in Fig. 2C. Two consecutive spikes (APs) occurring at short time interval (< 2 s) reached maximal signal amplitudes of 350% (normalized, Fig. 2C), which is less than the 600% reached by the “*1-AP* + *Plateau*” type of electrical signal (Fig. 2B). These data show that in the absence of any optical contamination (only one-cell present in the FOV), neurons generate the strongest Ca^2+^ transients during activity events involving plateau potentials (Fig. 2B, red dots). AP-Plateau waveforms in cultured cortical pyramidal neurons (Suppl. Figs. S3-B & S4-D) resembled the Ca^2+^-spikes described in cortical pyramidal cells of brain slices^41,42^, and were invariably potent initiators of strong optical transients in the Ca^2+^-imaging channel (Suppl. Fig. S3-A, green trace). The amplitude of the Ca^2+^ optical signal was proportional to the duration of the plateau. For example, in Suppl. Fig. S5, the increased duration of the events #2, #3 & #4 detected in the electrical channel, causes a corresponding amplitude-increase in the optical channel, despite each electrical event comprising the same number of APs (only 1 AP). Overall, these data indicate that it would be inaccurate to assume that in Ca^2+^ imaging experiments, the optical signal amplitude is reliably proportional to the number of APs neurons fire in a rapid sequence (Fig. 1B). Our dual electrical (whole-cell) and optical (Ca^2+^ imaging) monitoring of spontaneous neuronal activity showed that a significant number of high-amplitude Ca^2+^ optical signals are caused by an electrical event combining a depolarization plateau with only one AP, or several APs (AP-Plateau) (Suppl. Figs. S3-S6).

#### The Ca-imaging method captures subthreshold electrical depolarizations devoid of APs

In the next experimental series (n = 10 neurons), we reduced the concentration of the Ca^2+^-dye OGB1 to 25 μM. Single-cell Ca^2+^-imaging paired with whole-cell recording revealed robust Ca^2+^ transients coinciding with subthreshold depolarizations of the cell body, which lack any APs (Fig. 2D_3_ SE). Specifically, in 6 out of 10 cells, we optically detected 64 SEs devoid of APs. In the absence of an AP, the sustained depolarization (~ 20 mV amplitude, ~ 500 ms duration) was sufficient for the optical signal to emerge above noise (Fig. 2D_3_, asterisk). In some cases, even smaller than 20 mV and shorter than 500 ms subthreshold electrical events triggered conspicuous Ca^2+^ signals (Suppl. S7-B,C). Activation of Ca^2+^-permeable glutamate receptors was unnecessary, as somatic Ca^2+^ signals can be evoked by simple depolarization via injection of current (40—80 pA) through the patch pipette (Suppl. S8-A). Interestingly, in 3 cells, we recorded 5 instances where an inadvertent IPSP in the whole-cell channel reduced the amplitude of the current-injection-evoked somatic Ca^2+^ signal (Suppl. S8-B, asterisk).

To summarize, without concurrent electrical recordings (Suppl. Figs. S1-S8), it would be challenging to discern between three types of Ca^2+^ activity optical transients: [i] those triggered by trains of simple APs without underlying plateaus; [ii] those initiated by AP trains occurring alongside plateau depolarizations; and [iii] those triggered by subthreshold plateau depolarizations without any APs. This level of distinction (with- vs. without plateau) may be achievable in voltage imaging mode^15,17^.

### Voltage imaging

**Figure 3:**
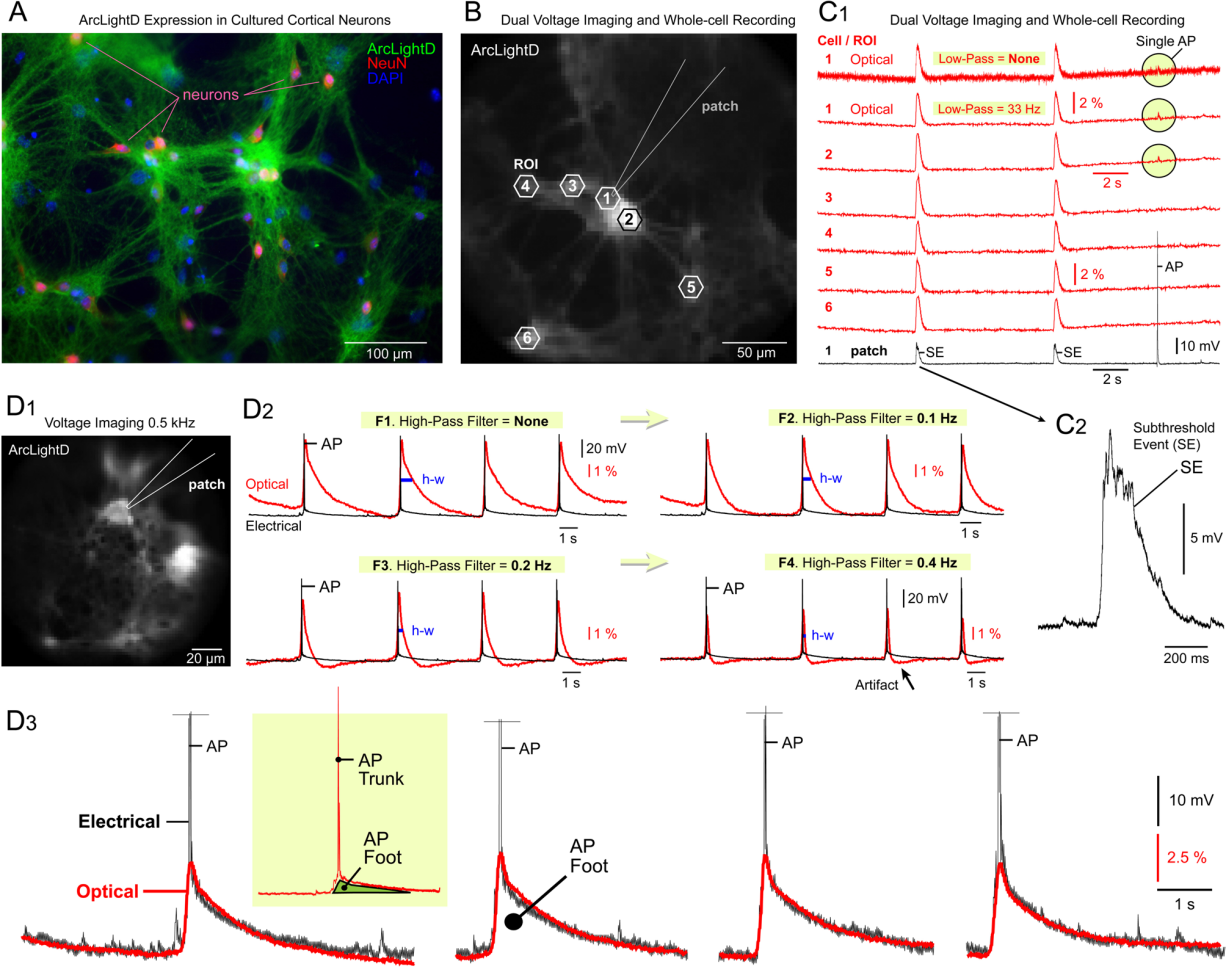
Combined GEVI imaging and whole-cell recordings. (**A**) Thy1-positive pyramidal neurons transduced with AAV_ArcLightD and counter-stained with neuronal marker (NeuN) and cell-nucleus marker (DAPI). (**B**) Voltage imaging conducted at a 2 ms sampling interval (0.5 kHz) – one video frame is shown. (**C**_**1**_) Optical trace from ROI #1 is displayed without a low-pass filter (top), and then with a low-pass digital filter (cutoff at 33 Hz). ROIs 2 – 6 are filtered. The trace labeled “patch” represents a whole-cell recording from Cell #1. A current-evoked action potential (“AP”) is visible only in optical traces from ROIs #1 and #2. (**C**_**2**_) A spikeless subthreshold electrical event (SE) shown on a finer scale. (**D**_**1**_) Same experimental setup as in panel *B*, but with a different coverslip and a 40 × lens. (**D**_**2**_) Dual recording of electrical (black) and optical (red) signals from a patched neuron. Four filter sets (F1 to F4) are applied to the same optical trace to demonstrate the impact of a digital high-pass filter on signal waveform. The frequency cutoffs for F1 to F4 are: None, 0.1 Hz, 0.2 Hz, and 0.4 Hz, respectively. Optical signal duration at half amplitude (half-width, h-w) changes with a stronger filter setting (from F1 to F4). A black arrow marks a filtering artifact that may resemble hyperpolarization. (**D**_**3**_) The pair of electrical (black) and optical (red) traces from the same neuron are amplitude-scaled to illustrate that ArcLightD tracks the slow component of the membrane potential change. Inset: The slow component of the electrical signal (green triangle) is termed the “*Foot of the AP*”, while the fast component of the action potential is termed the “*AP Trunk*”.

In the next experimental series, we transduced cultured cortical neurons with the voltage indicator, ArcLightD (Fig. 3A). ArcLight (and its derivatives Bongwoori, Marina, ArcLight-MT, ArcLight-ST) is one of the most versatile GEVIs, owing to its robust expression in neurons, exceptional brightness, consistent responses to sensory inputs over prolonged periods of chronic imaging, and substantial optical signal in vivo^18,43–48^.

#### Voltage-indicator ArcLightD responds strongly to plateau potentials

In pyramidal neurons expressing ArcLightD, we observed spontaneous neuronal activity using a voltage-imaging technique^26^. Optical signals from 7 neurons prominently featured plateau potentials (Suppl. Figs. S3-CD, Events #1 & #3), while single APs elicited weaker optical responses (Event #2). ArcLightD localized to the neuronal plasmalemma of dendrites and axons (Fig. 3A, ArcLightD). As a result, the neuronal cell bodies (Fig. 3A, NeuN) were connected by bundles of fluorescent neurites. Voltage imaging at 2 ms-interval (500 Hz) often unveiled similar optical transients across multiple ROIs (Fig. 3B,C), indicating synchronized depolarizations occurring within networks of cultured neurons. Evidently, in cell culture (current study), neighboring cortical neurons undergo highly correlated large-amplitude, long-lasting subthreshold depolarizations, mirroring the behavior observed in cortical neurons in vivo^18,49, 50^. Low-pass filtering did not significantly impact the amplitude of optical signals (Fig. 3C_1_, compare “*Low-Pass* = *None*” vs. “*Low-Pass* = *33 Hz*”), suggesting a lack of fast optical transients associated with sodium APs, for instance. Comparison of electrical (patch) and parallel optical (voltage imaging) traces obtained from the same cell (Fig. 3C_1_, Cell #1), revealed the subthreshold nature of the underlying electrical transients (SE). Electrical depolarizations generating robust ArcLightD signals frequently exhibited plateau depolarizations (10–20 mV), with durations exceeding 200 ms at half amplitude (Fig. 3C_2_). Firing of a single AP was detected in two cells: Cell #1 (which was patched), and Cell #2 which was adjacent to Cell #1 (Fig. 3C_1_). It is likely that the neurites of Cell #1 extended into the ROI of Cell #2, contributing their optical signals to both ROIs, #1 and #2. These findings suggest that the strongest optical signals in ArcLightD imaging are associated with plateau depolarizations.

While low-pass filtering minimally affected slow voltage waveforms (Fig. 3C_1_, 33 Hz cut-off), the high-pass filtering (digital filter type = ”RC” or “Median”) caused notable distortions of the optical signal duration (Fig. 3D_1-2_ red trace). High-pass filters significantly reduced the half-width of optical transients (Fig. 3D_1-2_, h-w), and negative transient distortions emerged (Fig. 3D_2_, black arrow), potentially leading to misinterpretations as afterhyperpolarizations in neurons. The key takeaway here is that digital high-pass filtering improves the baseline (Fig. 3D_2_, compare “F1.*Filter* = *None*” vs. “F4.*Filter* = *0.4 Hz*”), but induces severe distortions in voltage waveforms, potentially leading to erroneous interpretations. Specifically, overfiltering of voltage-imaging data can produce false spikes and false hyperpolarizations.

#### ArcLightD primarily captures APs endowed with a depolarization foot

**Figure 4:**
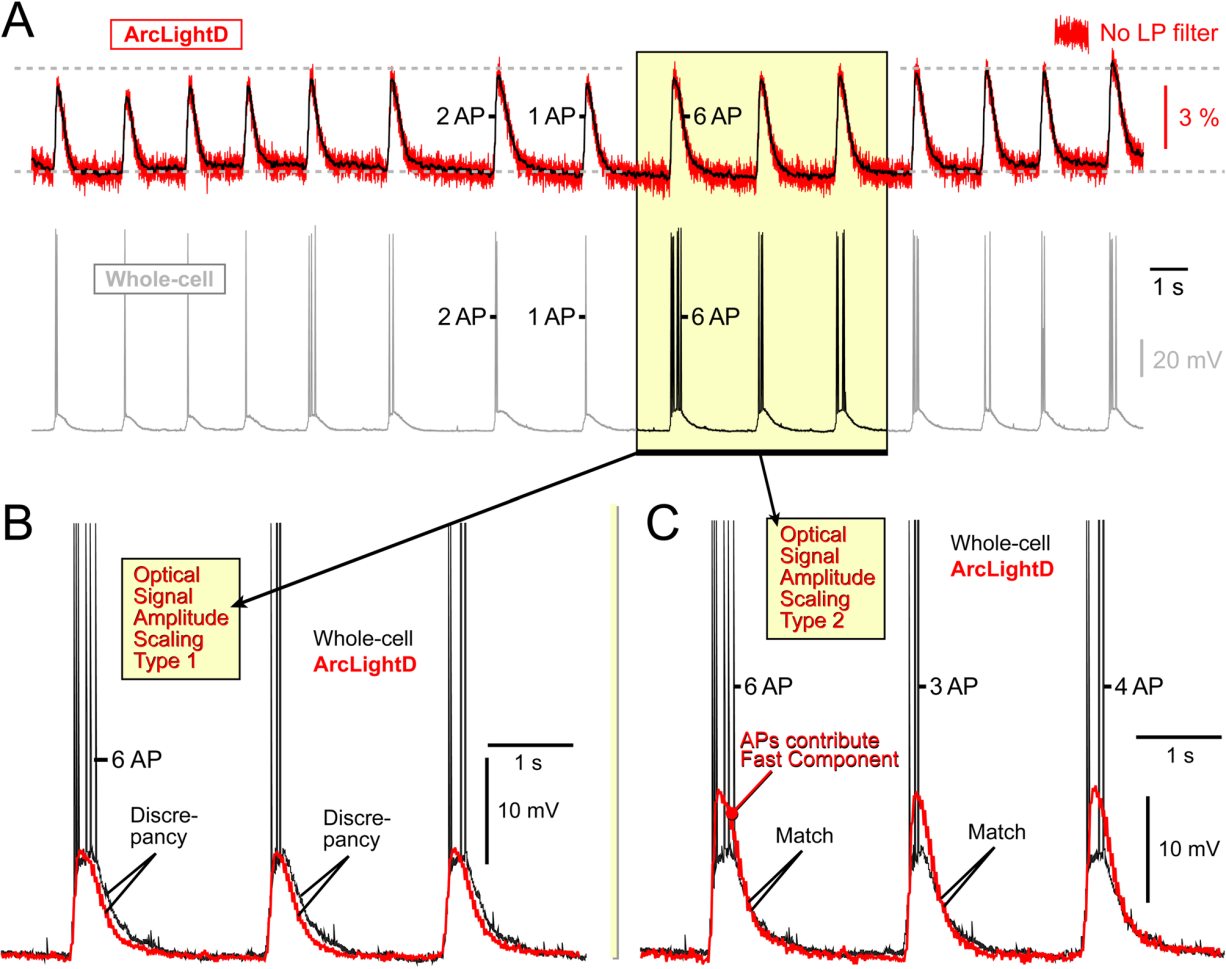
ArcLightD reports the slow phase of the electrical event. (**A**) Dual recording of electrical (whole-cell) and optical (ArcLightD) signals from a cultured neuron. The voltage imaging trace (top trace) is depicted without filtering (red) and with low-pass filtering (black optical trace). (**B**) A segment of the dual recording is presented here on a finer scale. The optical signal (red) is scaled to match the amplitude of the slow component of the electrical signal. The temporal discrepancy observed during the repolarization phase is labeled as “*Discrepancy*.” (**C**) Similar to panel *B*, but with different scaling; the optical signal is adjusted to match the decay of the electrical transient (*Match*). A portion of the optical signal extending beyond the peak of the plateau depolarization appears to integrate fast action potentials (APs) into a single optical transient (*Fast Component*).

Dual electrical and optical recordings from the same neuron suggested that ArcLightD responded strongly to the AP foot (Fig. 3D_3_, AP Foot), while the fast component of an AP (AP trunk) appeared to be too rapid for the indicator to detect. Additional evidence indicating that APs were not well represented in the ArcLightD optical signals came from neurons generating bursts of APs. Despite significant variability in the number of spikes within these bursts (Fig. 4A, Whole-cell), the corresponding ArcLightD optical signal amplitudes remained remarkably stable (Fig. 4A, ArcLightD, red trace). Optical transients caused by just 2 spikes (2 AP), or 6 consecutive spikes (6 AP), showed very similar amplitudes (Fig. 4A, compare “2 AP” vs. “6 AP”), suggesting that APs did not significantly contribute to the optical signal. Subsequently, we scaled down the optical signal (red) to match the amplitude of the slow component of the corresponding electrical transient (black), revealing a notable temporal discrepancy between the optical and electrical signals during the decaying (repolarization) phase of the event (Fig. 4B, *Discrepancy*). Adjusting the amplitude scaling factor to match the decaying phases of the optical (red) and electrical (black) transients produced a better match in voltage time course (Fig. 4C, Match), indicating that ArcLightD optical response was primarily driven by the slow component of the electrical signal: long-lasting plateau depolarization at the base of the AP burst. Despite expectations that the portion of the optical signal above the plateau (Fast Component) would grow proportionally to the number of APs in the burst (Fig. 4, 1-AP, 2-AP, or 6-AP), this was not observed in dual-recording experiments (Whole-cell and ArcLightD) performed on 5 neurons at 500 Hz, and 3 neurons using a 1000 Hz optical sampling rate. The fast component of the electrical signal, represented by the burst of APs riding on top of the plateau, contributed weakly to the ArcLightD optical signal, suggesting that ArcLightD integrated multiple APs into one compound optical transient (Fig. 4C, Fast Component). Invariably, the presence of a plateau notably enhanced the voltage optical signal (Suppl. Fig. S9). We conclude that ArcLightD exhibits a strong response to plateau depolarizations.

#### Comparisons between Ca^2+^ and voltage imaging traces

**Figure 5:**
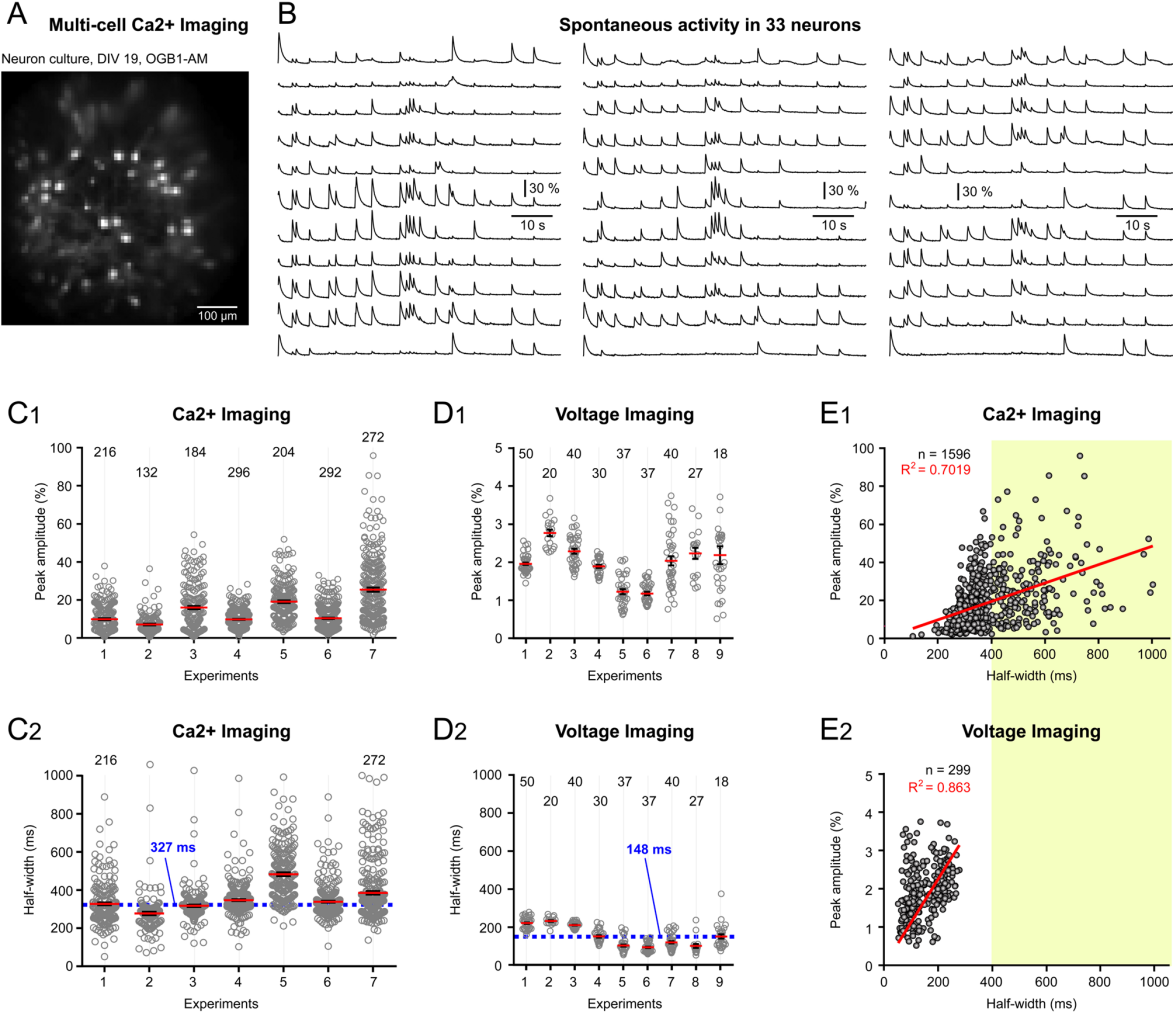
Multi-cell imaging of spontaneous activity – Comparisons between calcium and voltage transients. (**A**) Cultured cortical neurons, extracellularly loaded with the Ca^2+^-sensitive dye OGB1-AM. (**B**) Optical traces from 33 neurons depict ongoing spontaneous neuronal activity. (**C**_**1**_) Ca^2+^ imaging: Optical signal amplitudes from 7 neuronal cultures, each containing 8–10 neurons. Each data point represents one optical transient, and the number of data points per experiment (n) is indicated above each column. (**C**_**2**_) Same Ca^2+^ imaging data as in *C*_*1*_, with signal duration (half-width) quantified. The dashed horizontal line marks the median value of 327 ms. (**D**_**1**_** & D**_**2**_) Similar to panel *C*, but using ArcLightD as the optical indicator. The optical transient’s half-width median value now is only 148 ms. (**E**_**1**_) Optical transient’s amplitude plotted against the optical transient’s duration. Each data point represents one physiological event (n = 1,596 Ca^2+^ transients, from 7 cultures). (**E**_**2**_) Similar to panel *E*_*1*_, but using ArcLightD as the optical indicator (n = 299 depolarization (voltage) transients, from 9 cultures). Yellow shaded area highlights signal durations that are typical in Ca^2+^ imaging but are not typically observed in voltage imaging.

During episodes of unprovoked, spontaneous neuronal electrical activity, optical recordings allowed us to quantify numerous individual Ca^2+^ and voltage transients obtained from multiple ROIs within the same field of view (Fig. 5A,B). The average amplitudes of spontaneous Ca^2+^ transient (mean ± sem) per cell culture (per experiment, n = 7) are shown in Fig. 5C_1_, overlaid with the amplitudes of all recorded individual transients (open circles). Specifically, we analyzed 8–10 neurons per cell culture, over a period of 72 s, yielding between 132 and 296 transients (Fig. 5C_1_, “n” indicates the number of optical transients from one cell-culture). The total number of transients from all 7 experiments combined was 1,596. The average optical signal amplitude was 14.25 ± 0.2% ΔF/F (n = 1,596). The same data were also quantified for the duration of the Ca^2+^ transient measured at half amplitude (Fig. 5C_2_). The average optical signal half-width was 358.8 ± 3.1 ms (n = 1,596). The median half-width in these Ca^2+^ imaging sessions was 327 ms, marked by a horizontal dashed line (Fig. 5C_2_).

#### Comparisons of the voltage-imaging (ArcLightD) and Ca^2+^-imaging (OGB1-AM)

Due to the difference in speed between voltage and Ca^2+^ transients, these two imaging modalities were expected to produce markedly different reports of spontaneous network activity. To test this hypothesis, we characterized the ArcLightD data based on two parameters: (i) optical signal amplitude and (ii) duration (half-width). Using a 500 Hz sampling rate, we analyzed 6–10 neurons per cell-culture. Each recording session lasted 30 s, yielding between 18 and 50 voltage transients per cell culture (Fig. 5D_1_, “n” shown above the column of points indicates the number of optical transients detected). The total number of transients from all 9 voltage imaging experiments combined was 299. In these ArcLightD imaging sessions, the average peak amplitude of the optical signal ranged between 2 and 3% ΔF/F (Fig. 5D_1_), approximately 10 times smaller than in the Ca^2+^-imaging sessions on the same type of biological preparation (Fig. 5C_1_). Furthermore, the average optical signal half-width in the ArcLightD imaging sessions was 153.9 ± 3.5 ms (n = 299). The median half-width in the voltage imaging sessions was only 148 ms (Fig. 5D_2_, horizontal dashed line); approximately half of that recorded in the Ca^2+^ data (Fig. 5C_2_).

Having both amplitude and duration values for each optical transient in the experiment allowed us to generate *Amplitude vs. Duration* plots for both Ca^2+^-imaging (Fig. 5E_1_) and voltage-imaging results ((Fig. 5E_2_). We anticipated a linear dependence of Ca^2+^ transients on their duration, as the intracellular Ca^2+^ accumulates with repetitive spiking^9,51, 52^. On the other hand, we anticipated a sigmoidal distribution of the voltage transients, as the membrane-voltage changes more rapidly and has a narrower amplitude range than Ca^2+^ (Suppl. Figs. S3 and S9). A linear fit through the combined optical imaging data produced a coefficient of correlation of 0.7019 for Ca^2+^ optical transients (Fig. 5E_1_) and an even higher value (0.863) for the voltage optical transients (Fig. 5E_2_). These results suggest that optical transients in both Ca^2+^ and Voltage modalities share one important feature: regardless of the modality, *Ca*^*2*+^ or *Voltage*, during spontaneous neuronal activity, the optical transient amplitude is in good correlation with the duration of the underlying physiological event. Longer electrical transients (sustained depolarizations) generate larger-amplitude optical transients in both imaging modalities.

## Discussion

To elucidate the electrical events underlying optical signals in Ca^2+^-imaging and voltage-imaging techniques, we conducted dual-channel recordings from individual neurons in culture. One channel utilized whole-cell recording via a patch electrode attached to the cell body, while the other channel entailed optical recording from multiple pixels encompassing the patched neuron's cell body. This study yielded four outcomes: (1) Spikeless subthreshold depolarizations (plateaus) produced optical transients; (2) Plateaus strongly enhanced the AP optical signals; (3) The amplitude of the optical signal exhibited a strong and near-linear dependence on the duration (half-width) of the underlying electrical transient (Fig. 5); and (4) These findings, particularly points “1”, “2” and “3,” were consistent for both Ca^2+^-imaging (utilizing the Ca^2+^-sensitive dye OGB1) and voltage-imaging (employing a genetically encoded voltage indicator named ArcLightD).

Neuronal culture serves as a valuable experimental model for investigating fundamental concepts in neurobiology^53,54^, evaluating new drugs^55^, studying mechanisms of disease^56,57^; and developing experimental methods^17,58, 59^. Cell cultures have been instrumental in demonstrating that dual whole-cell and voltage-imaging recordings with the fastest performing GEVIs consistently reveal a small overestimation of the apparent AP kinetics^60,61^. Due to its very rapid rise and decay, the AP is the most kinetically demanding type of neuronal electrical signaling. Consequently, not only does the notoriously slow ON rate of various GEVI molecules influence the AP waveform, but even minor errors in glass pipette capacitance compensation, or series resistance, during whole-cell measurements can lead to significant overestimates of AP duration in various neuronal compartments, including the cell body, proximal axon, and axon boutons^54,60–62^. Another key advantage of cultured neurons is their ability to form synaptic connections and generate electrical activity in the absence of any stimulation^2,63^. Spontaneous (unprovoked) electrical activity, characteristic of both developing and mature brains^64^, was utilized in this study to assess the electrical signals underlying optical signals in Ca^2+^-imaging and voltage-imaging sessions (Figs. 1–5).

During multi-cell optical recordings, optical transients are driven by neuronal electrical signaling comprised of APs and dendritic plateau potentials^65^. While EPSP-mediated Ca^2+^ transients are observable in two-photon Ca^2+^ imaging from individual dendritic spines^66,67^, they do not manifest in wide-field imaging^68^. In large-scale imaging applications, it is common practice to interpret optically-detected neuronal Ca^2+^ transients as neuronal action potentials (APs), or to not explicitly state the underlying electrical signal associated with the reported Ca^2+^ transient^32,34, 35^. It is important to note that in in vivo Ca^2+^ recordings, the probability of detecting a single action potential (1 AP) occurring in the neuronal cell body is only “0.1”, as demonstrated in their supplementary figure #23^35^. Additionally, the detection rate for two successive action potentials (2 AP) in the Ca^2+^ -imaging channel is typically lower than “0.4”. Through dual electrical-optical recordings from the same neuron, it has been observed that 6 out of 10 electrical events involving 2 consecutive action potentials resulted in no detectable changes in the Ca^2+^-imaging channel^35^. Smetters et al.^9^, their figure 2A, suggested that subthreshold depolarizations and/or excitatory postsynaptic potentials (EPSPs) that fail to reach the threshold for triggering an AP regularly do not generate detectable calcium transients^9^. Twenty years later, new Ca^2+^ indicators combined with sensitive imaging equipment (two-photon imaging) have run into the same problem—a subthreshold depolarization cannot be detected in the Ca^2+^-imaging channel^18^, their figure 5. However, our data reveal that subthreshold depolarizations exceeding certain thresholds (> 10 mV in amplitude, and > 100 ms in duration) do produce detectable calcium transients (Fig. 1), see also^1^, their figure 4; see also^69^, their figure 8.

In our study, successive Ca^2+^ transients obtained from the same neuron exhibited significant amplitude variability (Fig. 1, and Suppl. Figs. S3 thru S6), consistent with previous findings^1,2^. This variability is attributed to the slow OFF rate of the Ca^2+^ indicator (OGB1-AM or GCaMP6f), which integrates photons from fast successive events^70^. The OFF rate of the voltage indicator ArcLightD is approximately 3 times faster^28^, and hence ArcLightD was expected to produce less amplitude variability (less optical signal integration) in optical traces. However, this was not the case. The amplitudes of optically-recorded voltage transients (Suppl. Figs. S3-CD and S9) were as variable as the amplitudes of the optically-recorded Ca^2+^ transients (Suppl. Figs. S3 thru S6). ArcLightD is known to activate and deactivate more slowly than the newer GEVI variants QuasAr6, VARNAM, Archon1, or ASAP4^28,59, 60, 71^, which may contribute to its “integrative property”. In summary, three indicators OGB1-AM, GCaMP6f, and ArcLightD are relatively “slow”, exhibiting a linear relation between the optical signal amplitude and optical signal duration in both *Ca*^*2*+^*-imaging* (Fig. 5E_1_) and *voltage-imaging* experiments (Fig. 5E_2_). While this may be a disadvantage for detecting individual APs, it may be a crucial advantage for detecting plateau depolarizations^72^.

Previously, it was suggested that there is a strong correlation between APs and somatic increases in Ca^2+^^9^. However, our data indicate that this notion is only partially accurate. We observed frequent occurrences of distinctive electrical waveforms combining: (a) fast sodium spikes (APs) with (b) prolonged plateau depolarizations, which we term ‘AP-Plateaus’. These AP-Plateau electrical waveforms observed in cultured cortical pyramidal neurons resemble the AP bursts induced by “Ca^2+^-spikes” described in cortical pyramidal cells of brain slices^41,42^. We found that *AP-Plateau* electrical signals consistently initiated robust optical transients in both the *Ca*^*2*+^*-imaging* channel (Suppl. Fig. S3-AB) and the *voltage-imaging* channel (Suppl. Fig. S3-CD). In complex electrical events comprising a slow depolarization (AP foot) and a rapid sodium AP (AP trunk), ArcLightD appeared to mirror the waveform of the 'slow foot' rather than that of the 'fast trunk' (Fig. 3D_3_). One-second-long neuronal depolarizations detected by ArcLightD in the current study (Fig. 4A, red trace) are comparable to visually-evoked neuronal UP states detected in vivo using a derivative of ArcLightD^18^, suggesting that sensory-evoked neuronal UP states are electrically characterized by AP bursts riding on top of 1–2-s-long plateau depolarizations (Fig. 4C, black trace). To summarize, AP-Plateau events significantly surpass simple APs as inducers of optical transients. This observation holds true for both calcium-imaging and voltage-imaging experiments.

In in vivo Ca^2+^ imaging studies, the reported neuronal activity likely consists of a mixture of three signal types: [a] simple AP firing without plateau (Suppl. Fig. S2), [b] AP firing with underlying plateau (Suppl. Fig. S3), and [c] plateau depolarizations devoid of any APs (Fig. 2 and Suppl. Fig. S7). Differentiating between these 3 categories of electrical signaling may be important for understanding the relationship between neuronal electrical signals and animal behavior^73^. These three categories of electrical signaling produce similar Ca^2+^ transients but lead to vastly different circuit modifications. For example, APs with plateau^74^ have been shown to induce ‘*place preference*’ in CA1 cells, whereas APs without plateau are ineffective on the task of place preference^74^.

In our study, we compared the practicality of three optical indicators, OGB1-AM, GCaMP6f, and ArcLightD, for monitoring spontaneous electrical activity in cortical neuron cultures. While these indicators demonstrated the ability to monitor activity in numerous cells simultaneously across multiple recording trials (each lasting 30 s or more), the Ca^2+^ imaging method exhibited several advantages. Firstly, it resulted in healthier neurons with more abundant spontaneous activity (> twofold greater frequency, not shown) and stronger optical signals (> tenfold greater ΔF/F, Fig. 5). Additionally, Ca^2+^ imaging allowed for reduced intensity of illumination (excitation light), minimizing photobleaching and photodamage, low sampling speeds without a significant loss of physiological information, prolonged viability of biological preparations, and generated smaller data files.

While considering comparisons between ArcLightD and Ca^2+^-indicator, it is important to note that ArcLightD is entirely based on membrane depolarization, while Ca^2+^ signal may draw amplitude from three complementary sources: voltage-gated Ca^2+^ channels, Ca^2+^-permeable glutamate receptors, and most importantly, release from internal stores of Ca^2+^^30^. Despite the aforementioned considerations, ArcLightD demonstrated certain advantages over Ca^2+^ indicators. Specifically, it consistently produced faster (narrower) optical transients, potentially enabling the differentiation of simple APs from complex electrical signals termed AP-Plateaus (AP bursts with underlying plateau depolarization, as shown in Suppl. Figure 5D). Moreover, ArcLightD allowed for more accurate estimates and quantifications of depolarization half-width, providing valuable information about the duration of time a neuron has spent in the depolarized-UP state^18^. This information was not available in OGB1-AM and GCaMP6f measurements due to the slow OFF rate of the calcium indicator (as depicted in Suppl. Fig. S3-A, green trace, events #3 and #4; see also Suppl. Fig. S6-B, events #1 and #2).

In summary, our study highlights the importance of understanding the electrical events underlying optical signals in both Ca^2+^ and voltage imaging techniques, shedding light on the dynamics of neuronal activity in cultured neuronal networks.

## Methods

### Ethics statements

All experiments were performed in accordance with relevant guidelines and regulations. The use of newborn mice for preparation of cell cultures was approved by the UConn Health Institutional Animal Care and Use Committee (IACUC), animal protocol (#200902). The study is reported in accordance with ARRIVE guidelines.

### Primary cortical neuronal cell culture

Newborn pups (P0-P1) of C57BL/6 mice were anesthetized by hypothermia and decapitated. Cortices were isolated in dissecting medium (DM) consisting of the following: HBSS without calcium and magnesium (Gibco, Thermo Fisher Scientific, USA) supplemented with sodium pyruvate 1 mM, glucose 0.02% (w/v), and HEPES 10 mM. Cortices were washed three times in DM, followed by enzymatic digestion at 37 °C in the water bath in DM with trypsin (1 mg/ml freshly dissolved before this step; Sigma-Aldrich, USA), for 20 min with occasional mixing. After digestion, loosened cortices were left to settle on the bottom of the tube. They were carefully washed three times in DM, and additional three times in temperature-equilibrated plating medium (PM) consisting of Dulbecco's Modified Eagle Medium/Nutrient Mixture F-12 (Gibco, Thermo Fisher Scientific) supplemented with FBS 10%, glucose 0.02% (w/v), gentamycin 10 µg/ml. Finally, the loosened tissue was carefully mechanically digested in 1 ml of PM with a 1000 µl pipette tip (up to 20 strokes, avoiding bubbles) and was left for 5 min at room temperature, allowing bigger pieces of tissue to settle on the bottom of the tube. The cell suspension (without bigger pieces of tissue) was used for plating onto 12 mm round glass coverslips (coated with poly-l-ornithine 50 μg/ml) in 24-well plates (∼60,000 cells/well). The medium was changed to maintenance medium (MM) within 2 h after the plating. MM consisted of BrainPhys Neuronal Medium (STEMCELL Technologies, USA) supplemented with SM1 Neuronal Supplement (STEMCELL Technologies) 2%, GlutaMAX 2 mM, and gentamycin 10 µg/ml. The following day, half of the medium was changed with fresh MM. Three days after plating, half of the medium was changed with fresh MM with cytosine β-d-arabinofuranoside hydrochloride (final concentration, 1–3 μM; Sigma-Aldrich). Half of the medium was changed with fresh MM every third day. Neurons were imaged after DIV11, at which point they were mature enough to generate action potentials.

### Immunostaining

Phosphate buffer solution (PBS, 0.01 M, pH 7.4) contained: 8 mM Na2HPO4, 2 mM NaH2PO4 × 2 H2O, and 147 mM NaCl. Coverslips containing cultured neurons were washed in PBS and fixed in 4% PFA for 30 min. Next, they were washed in PBS three times for 10 min and incubated in the blocking solution containing 10% bovine serum albumin (BSA) and 0.01% Triton-X 100 in PBS for 1 h. After the blocking step, cells were incubated with the primary mouse anti-NeuN antibody (1:100, MillliporeSigma, USA) in PBS overnight at 4 °C. Cells were washed in PBS 3 times for 10 min and incubated with secondary goat anti-mouse Alexa Fluor 555 antibody (1:200, Invitrogen, USA) for 2 h at room temperature. After washing in PBS 3 times for 10 min, cells were stained with nuclear counterstain Hoechst 3342 (1 μg/ml) for 10 min, washed 4 times for 5 min in PBS and mounted using MOWIOL medium. Images of immunolabeled primary cortical neurons were acquired using Keyence: BZ-X800 microscope (Keyence, Corporation of America, IL, USA) equipped with DAPI (OP-87762), GFP (OP-87763) and TexasRed (OP-87765) filter sets.

### Neuron labeling with OGB1-AM

Primary cortical neurons seeded on glass coverslips were loaded with membrane-permeable 2.67 μM OGB1-AM (Invitrogen, USA) for 30 min. Dye stock solution was prepared as described in the manufacturer’s instructions. Upon washing the dye, coverslips were incubated at 37 °C for 10 min in MM, and transferred to the recording chamber on the microscope for imaging.

### Neuron labeling with ArcLightD

Viral vector AAV1-hsyn-ArcLightD^28^ (ArcLight A242, Addgene #100037) was kindly provided by Vincent Pieribone (Yale University, New Haven, CT, USA). The AAV stock solution contained 0.25 × 10^13 particles dissolved in culture-media. Primary cortical neurons were transduced by adding 1 μL of the AAV stock solution to the wells with neuronal cells (volume = 800 μL) on day-in-vitro 4–8 (DIV4–8) and imaged at least 7 days following the transduction. On the first day of transduction, the final concentration of AAV in the well was estimated to be 0.3 × 10^10 particles.

### Optical recordings

Coverslips with labeled neurons were transferred to a recording chamber of an Olympus BX51WI microscope. The recording chamber was perfused with warm (34 ± 1 °C) ACSF (in mM): 125 NaCl, 26 NaHCO3, 10 glucose, 5 KCl, 1.26 KH2PO4, 1.2 CaCl2 and 2 MgSO4 (pH 7.4, osmolality ~ 300 mOsm/kg), continuously bubbled with 95% O_2_ and 5% CO_2_. OGB-1 and ArcLightD, were excited using a 470 nm light emitting diode, LED (pE, CoolLED, Andover, UK), excitation filter of 480/40 nm, dichroic filter of 510 nm, and emission filter of 535/50 nm. Max power of the LED illumination at 100% was 14 mW/mm^2^. For Ca^2+^ imaging at 14 Hz rate we used only 10% of the illumination power. Images were projected onto 80 × 80-pixel CCD camera (NeuroCCD-SMQ; RedShirtImaging, Decatur, GA, USA) using 10x, 0.3 NA and 40 × 0.8 NA water immersion objectives. In the case of calcium imaging, images were sampled at 72 ms per frame (~ 14 Hz) or 2 ms per frame (500 Hz). For voltage imaging, images were sampled at 2 ms per frame (500 Hz) unless otherwise indicated. Optical data were analyzed using Neuroplex software (RedShirtImaging, Decatur, GA, USA).

### Whole-cell recordings

Patch pipettes (5–7 MΩ) were filled with an intracellular solution containing (in mM) 135 potassium gluconate, 10 HEPES, 2 MgCl2, 3 Mg-ATP, 0.3 GTP-tris, and 10 phosphocreatine (pH 7.2 adjusted with KOH, osmolality ~ 295 mOsm/kg). Electrical signals were amplified using the MultiClamp 700B and digitized simultaneously with two input boards: (i) Digidata Series 1400A (Molecular Devices, Union City, CA, USA) at 5 kHz, and (ii) Neuroplex (RedShirtImaging, Decatur, GA, USA) at 0.5—1 kHz. The capability of Neuroplex to embed electrical recordings within the optical data file is particularly advantageous for swiftly comparing electrical and optical signals in the same display window. Optical responses to the changes in membrane voltage were simultaneously recorded with NeuroCCD camera (NeuroCCD-SMQ; RedShirtImaging, Decatur, GA) connected to the microscope via 0.67 × demagnifier using a 40x, 0.8 NA water immersion objective. For intracellular injections of the Ca^2+^-sensitive dye, Oregon Green 488 BAPTA-1, hexapotassium salt (OGB1, Invitrogen, USA), was dissolved in intracellular solution at 2 concentrations: 100 μM and 25 μM. In figures, patch electrode traces containing APs are displayed unfiltered, while traces without APs are filtered with a low-pass 400 Hz cut-off.

### Data analysis

Optical traces were conditioned and analyzed using Neuroplex and Clampfit software. Analysis of optical data, including spatial averaging, exponential subtraction, and filtering, was conducted with the Neuroplex data acquisition and analysis software (RedShirtImaging, Decatur, GA, USA). The values for low-pass Gaussian filter and high pass RC (or Median) filter are stated in the figures. Averaged intensity of the region of interests (ROIs) covering neuronal cell bodies were used for analysis. Optical signal amplitudes are expressed as ΔF/F, where F represents the resting fluorescence intensity at the beginning of the optical trace (baseline), and ΔF represents the intensity change from the baseline fluorescence during a spontaneous electrical activity event. Amplitude and half-width of spontaneous activity events were determined using Clampfit software (version 10.2.2. for Windows, Molecular Devices, San Jose, CA, USA). The criteria used to delineate simple action potentials (APs) from AP-Plateaus in patch clamp recordings were based on the presence of depolarizing potentials at the base of the spike; greater than 10 mV in amplitude and longer than 100 ms in duration. To determine if an electrically-recorded 1-AP event was able to induce Ca^2+^ optical signal, the optical threshold was set at 3 standard deviations above the baseline noise (96 “1-AP events” have met this criterion). To include APs into Fig. 2C (amplitude versus time interval), the optical threshold was set at 1 standard deviation above the baseline noise (138 “1-AP events” have met this criterion). Results are presented as average values ± the standard error of the mean (SEM), unless otherwise stated. Statistical analysis was not needed to reach the conclusions presented.

## Supplementary Information

Supplementary Figures.

## Data Availability

The datasets generated during and/or analyzed during the current study are available from the corresponding author on reasonable request.

## References

[1] Murphy, T. H., Blatter, L. A., Wier, W. G. & Baraban, J. M. Spontaneous synchronous synaptic calcium transients in cultured cortical neurons. *J. Neurosci.* **12**(12), 4834–4845 (1992). 1361198 10.1523/JNEUROSCI.12-12-04834.1992 PMC6575780

[2] Cohen, E., Ivenshitz, M., Amor-Baroukh, V., Greenberger, V. & Segal, M. Determinants of spontaneous activity in networks of cultured hippocampus. *Brain Res.* **1235**, 21–30 (2008). 18602907 10.1016/j.brainres.2008.06.022

[3] Antic, S. D., Zhou, W. L., Moore, A. R., Short, S. M. & Ikonomu, K. D. The decade of the dendritic NMDA spike. *J. Neurosci. Res.* **88**(14), 2991–3001 (2010). 20544831 10.1002/jnr.22444 PMC5643072

[4] Larkum, M. E., Nevian, T., Sandler, M., Polsky, A. & Schiller, J. Synaptic integration in tuft dendrites of layer 5 pyramidal neurons: A new unifying principle. *Science* **325**(5941), 756–760 (2009). 19661433 10.1126/science.1171958

[5] Antic, S. D., Hines, M. & Lytton, W. W. Embedded ensemble encoding hypothesis: The role of the “Prepared” cell. *J. Neurosci. Res.* **96**(9), 1543–1559 (2018). 29633330 10.1002/jnr.24240 PMC6095748

[6] Wilson, C. J. & Kawaguchi, Y. The origins of two-state spontaneous membrane potential fluctuations of neostriatal spiny neurons. *J. Neurosci.* **16**(7), 2397–2410 (1996). 8601819 10.1523/JNEUROSCI.16-07-02397.1996 PMC6578540

[7] Goto, Y. & O’Donnell, P. Network synchrony in the nucleus accumbens in vivo. *J. Neurosci.* **21**(12), 4498–4504 (2001). 11404437 10.1523/JNEUROSCI.21-12-04498.2001 PMC6762756

[8] Dana, H. *et al.* Sensitive red protein calcium indicators for imaging neural activity. *elife*10.7554/eLife.12727 (2016). 27011354 10.7554/eLife.12727 PMC4846379

[9] Smetters, D., Majewska, A. & Yuste, R. Detecting action potentials in neuronal populations with calcium imaging. *Methods* **18**(2), 215–221 (1999). 10356353 10.1006/meth.1999.0774

[10] Abdelfattah, A. S. *et al.* Sensitivity optimization of a rhodopsin-based fluorescent voltage indicator. *Neuron* **111**(10), 1547-1563 e9 (2023). 37015225 10.1016/j.neuron.2023.03.009 PMC10280807

[11] Evans, S. W. *et al.* A positively tuned voltage indicator for extended electrical recordings in the brain. *Nat. Methods* **20**(7), 1104–1113 (2023). 37429962 10.1038/s41592-023-01913-z PMC10627146

[12] Alich, T. C. *et al.* Bringing to light the physiological and pathological firing patterns of human induced pluripotent stem cell-derived neurons using optical recordings. *Front. Cell Neurosci.* **16**, 1039957 (2022). 36733665 10.3389/fncel.2022.1039957 PMC9887032

[13] Hochbaum, D. R. *et al.* All-optical electrophysiology in mammalian neurons using engineered microbial rhodopsins. *Nat. Methods* **11**(8), 825–833 (2014). 24952910 10.1038/nmeth.3000 PMC4117813

[14] Bando, Y., Sakamoto, M., Kim, S., Ayzenshtat, I. & Yuste, R. Comparative evaluation of genetically encoded voltage indicators. *Cell Rep.* **26**(3), 802-813 e4 (2019). 30650368 10.1016/j.celrep.2018.12.088 PMC7075032

[15] Walker, A. S. *et al.* Optical spike detection and connectivity analysis with a far-red voltage-sensitive fluorophore reveals changes to network connectivity in development and disease. *Front. Neurosci.* **15**, 643859 (2021). 34054405 10.3389/fnins.2021.643859 PMC8155641

[16] Piatkevich, K. D. *et al.* A robotic multidimensional directed evolution approach applied to fluorescent voltage reporters. *Nat. Chem. Biol.* **14**(4), 352–360 (2018). 29483642 10.1038/s41589-018-0004-9 PMC5866759

[17] Puppo, F. *et al.* All-optical electrophysiology in hiPSC-derived neurons with synthetic voltage sensors. *Front. Cell Neurosci.* **15**, 671549 (2021). 34122014 10.3389/fncel.2021.671549 PMC8193062

[18] Bando, Y., Wenzel, M. & Yuste, R. Simultaneous two-photon imaging of action potentials and subthreshold inputs in vivo. *Nat. Commun.* **12**(1), 7229 (2021). 34893595 10.1038/s41467-021-27444-9 PMC8664861

[19] Bullen, A. & Saggau, P. High-speed, random-access fluorescence microscopy: II. Fast quantitative measurements with voltage-sensitive dyes. *Biophys. J.* **76**(4), 2272–2287 (1999). 10096922 10.1016/S0006-3495(99)77383-2 PMC1300200

[20] Nadella, K. M. *et al.* Random-access scanning microscopy for 3D imaging in awake behaving animals. *Nat. Methods* **13**(12), 1001–1004 (2016). 27749836 10.1038/nmeth.4033 PMC5769813

[21] Villette, V. *et al.* Ultrafast two-photon imaging of a high-gain voltage indicator in awake behaving mice. *Cell* **179**(7), 1590-1608 e23 (2019). 31835034 10.1016/j.cell.2019.11.004 PMC6941988

[22] Weber, T. D., Moya, M. V., Kilic, K., Mertz, J. & Economo, M. N. High-speed multiplane confocal microscopy for voltage imaging in densely labeled neuronal populations. *Nat. Neurosci.* **26**(9), 1642–1650 (2023). 37604887 10.1038/s41593-023-01408-2 PMC11209746

[23] Adam, Y. *et al.* Voltage imaging and optogenetics reveal behaviour-dependent changes in hippocampal dynamics. *Nature* **569**(7756), 413–417 (2019). 31043747 10.1038/s41586-019-1166-7 PMC6613938

[24] Tanese, D. *et al.* Imaging membrane potential changes from dendritic spines using computer-generated holography. *Neurophotonics* **4**(3), 031211 (2017). 28523281 10.1117/1.NPh.4.3.031211 PMC5428833

[25] Zhu, M. H., Jang, J., Milosevic, M. M. & Antic, S. D. Population imaging discrepancies between a genetically-encoded calcium indicator (GECI) versus a genetically-encoded voltage indicator (GEVI). *Sci. Rep.* **11**(1), 5295 (2021). 33674659 10.1038/s41598-021-84651-6 PMC7935943

[26] Popovic, M. *et al.* Imaging submillisecond membrane potential changes from individual regions of single axons, dendrites and spines. *Adv. Exp. Med. Biol.* **859**, 57–101 (2015). 26238049 10.1007/978-3-319-17641-3_3 PMC5671121

[27] Piatkevich, K. D. *et al.* Population imaging of neural activity in awake behaving mice. *Nature* **574**(7778), 413–417 (2019). 31597963 10.1038/s41586-019-1641-1 PMC6858559

[28] Jin, L. *et al.* Single action potentials and subthreshold electrical events imaged in neurons with a fluorescent protein voltage probe. *Neuron* **75**(5), 779–785 (2012). 22958819 10.1016/j.neuron.2012.06.040 PMC3439164

[29] Mao, B. Q., Hamzei-Sichani, F., Aronov, D., Froemke, R. C. & Yuste, R. Dynamics of spontaneous activity in neocortical slices. *Neuron* **32**(5), 883–898 (2001). 11738033 10.1016/s0896-6273(01)00518-9

[30] Ross, W. N. Changes in intracellular calcium during neuron activity. *Annu. Rev. Physiol.* **51**, 491–506 (1989). 2653192 10.1146/annurev.ph.51.030189.002423

[31] Spellman, T., Svei, M., Kaminsky, J., Manzano-Nieves, G. & Liston, C. Prefrontal deep projection neurons enable cognitive flexibility via persistent feedback monitoring. *Cell* **184**(10), 2750-2766 e17 (2021). 33861951 10.1016/j.cell.2021.03.047 PMC8684294

[32] Liang, B. *et al.* Distinct and dynamic ON and OFF neural ensembles in the prefrontal cortex code social exploration. *Neuron* **100**(3), 700-714 e9 (2018). 30269987 10.1016/j.neuron.2018.08.043 PMC6224317

[33] Grodem, S. *et al.* An updated suite of viral vectors for in vivo calcium imaging using intracerebral and retro-orbital injections in male mice. *Nat. Commun.* **14**(1), 608 (2023). 36739289 10.1038/s41467-023-36324-3 PMC9899252

[34] Deneux, T. *et al.* Accurate spike estimation from noisy calcium signals for ultrafast three-dimensional imaging of large neuronal populations in vivo. *Nat. Commun.* **7**, 12190 (2016). 27432255 10.1038/ncomms12190 PMC4960309

[35] de Vries, S. E. J. *et al.* A large-scale standardized physiological survey reveals functional organization of the mouse visual cortex. *Nat. Neurosci.* **23**(1), 138–151 (2020). 31844315 10.1038/s41593-019-0550-9 PMC6948932

[36] Vogelstein, J. T. *et al.* Spike inference from calcium imaging using sequential Monte Carlo methods. *Biophys. J.* **97**(2), 636–655 (2009). 19619479 10.1016/j.bpj.2008.08.005 PMC2711341

[37] Mukamel, E. A., Nimmerjahn, A. & Schnitzer, M. J. Automated analysis of cellular signals from large-scale calcium imaging data. *Neuron* **63**(6), 747–760 (2009). 19778505 10.1016/j.neuron.2009.08.009 PMC3282191

[38] Tada, M., Takeuchi, A., Hashizume, M., Kitamura, K. & Kano, M. A highly sensitive fluorescent indicator dye for calcium imaging of neural activity in vitro and in vivo. *Eur. J. Neurosci.* **39**(11), 1720–1728 (2014). 24405482 10.1111/ejn.12476 PMC4232931

[39] Ait Ouares, K. & Canepari, M. The origin of physiological local mGluR1 Supralinear Ca(2+) signals in cerebellar Purkinje neurons. *J. Neurosci.* **40**(9), 1795–1809 (2020). 31969470 10.1523/JNEUROSCI.2406-19.2020 PMC7046445

[40] Wang, M. *et al.* Single-neuron representation of learned complex sounds in the auditory cortex. *Nat. Commun.* **11**(1), 4361 (2020). 32868773 10.1038/s41467-020-18142-z PMC7459331

[41] Williams, S. R. & Stuart, G. J. Mechanisms and consequences of action potential burst firing in rat neocortical pyramidal neurons. *J. Physiol.* **2**, 467–482 (1999). 10.1111/j.1469-7793.1999.00467.x PMC2269673 10581316

[42] Larkum, M. E., Zhu, J. J. & Sakmann, B. A new cellular mechanism for coupling inputs arriving at different cortical layers. *Nature* **398**(6725), 338–341 (1999). 10192334 10.1038/18686

[43] Platisa, J. *et al.* Voltage imaging in the olfactory bulb using transgenic mouse lines expressing the genetically encoded voltage indicator ArcLight. *Sci. Rep.* **12**(1), 1875 (2022). 35115567 10.1038/s41598-021-04482-3 PMC8813909

[44] Borden, P. Y. *et al.* Genetically expressed voltage sensor ArcLight for imaging large scale cortical activity in the anesthetized and awake mouse. *Neurophotonics* **4**(3), 031212 (2017). 28491905 10.1117/1.NPh.4.3.031212 PMC5416966

[45] Rhee, J. K., Iwamoto, Y. & Baker, B. J. Visualizing oscillations in brain slices with genetically encoded voltage indicators. *Front. Neuroanat.* **15**, 741711 (2021). 34795565 10.3389/fnana.2021.741711 PMC8592998

[46] Nakajima, R., Laskaris, N., Rhee, J. K., Baker, B. J. & Kosmidis, E. K. GEVI cell-type specific labelling and a manifold learning approach provide evidence for lateral inhibition at the population level in the mouse hippocampal CA1 area. *Eur. J. Neurosci.* **53**(9), 3019–3038 (2021). 33675122 10.1111/ejn.15177

[47] Storace, D. A. & Cohen, L. B. Measuring the olfactory bulb input-output transformation reveals a contribution to the perception of odorant concentration invariance. *Nat. Commun.* **8**(1), 81 (2017). 28724907 10.1038/s41467-017-00036-2 PMC5517565

[48] Platisa, J., Vasan, G., Yang, A. & Pieribone, V. A. Directed evolution of key residues in fluorescent protein inverses the polarity of voltage sensitivity in the genetically encoded indicator ArcLight. *ACS Chem. Neurosci.* **8**(3), 513–523 (2017). 28045247 10.1021/acschemneuro.6b00234 PMC5355904

[49] Poulet, J. F. & Petersen, C. C. Internal brain state regulates membrane potential synchrony in barrel cortex of behaving mice. *Nature* **454**(7206), 881–885 (2008). 18633351 10.1038/nature07150

[50] Volgushev, M., Chauvette, S., Mukovski, M. & Timofeev, I. Precise long-range synchronization of activity and silence in neocortical neurons during slow-wave sleep. *J. Neurosci.* **26**(21), 5665–5672 (2006). 16723523 10.1523/JNEUROSCI.0279-06.2006 PMC6675259

[51] Antic, S. D. Action potentials in basal and oblique dendrites of rat neocortical pyramidal neurons. *J. Physiol.* **550**(1), 35–50 (2003). 12730348 10.1113/jphysiol.2002.033746 PMC2343022

[52] Schiller, J., Helmchen, F. & Sakmann, B. Spatial profile of dendritic calcium transients evoked by action potentials in rat neocortical pyramidal neurones. *J. Physiol.* **487**(Pt 3), 583–600 (1995). 8544123 10.1113/jphysiol.1995.sp020902 PMC1156647

[53] Martinelli, D. C. *et al.* Expression of C1ql3 in discrete neuronal populations controls efferent synapse numbers and diverse behaviors. *Neuron* **91**(5), 1034–1051 (2016). 27478018 10.1016/j.neuron.2016.07.002 PMC5017910

[54] Ritzau-Jost, A. *et al.* Large, stable spikes exhibit differential broadening in excitatory and inhibitory neocortical boutons. *Cell Rep.* **34**(2), 108612 (2021). 33440142 10.1016/j.celrep.2020.108612 PMC7809622

[55] Rossetti, A. C., Koch, P. & Ladewig, J. Drug discovery in psychopharmacology: From 2D models to cerebral organoids. *Dialogues Clin. Neurosci.* **21**(2), 203–224 (2019). 31636494 10.31887/DCNS.2019.21.2/jladewig PMC6787544

[56] Elamin, M., Lemtiri-Chlieh, F., Robinson, T. M. & Levine, E. S. Dysfunctional sodium channel kinetics as a novel epilepsy mechanism in chromosome 15q11-q13 duplication syndrome. *Epilepsia*10.1111/epi.17687 (2023). 37329181 10.1111/epi.17687 PMC10529833

[57] Kapadia, M. *et al.* Effects of sustained i.c.v. infusion of lupus CSF and autoantibodies on behavioral phenotype and neuronal calcium signaling. *Acta Neuropathol. Commun.* **5**(1), 70 (2017). 28882191 10.1186/s40478-017-0473-1 PMC5590168

[58] Monakhov, M. V. *et al.* Screening and cellular characterization of genetically encoded voltage indicators based on near-infrared fluorescent proteins. *ACS Chem. Neurosci.* **11**(21), 3523–3531 (2020). 33063984 10.1021/acschemneuro.0c00046

[59] Bloxham, B., Brinks, D., Kheifets, S. & Cohen, A. E. Linearly polarized excitation enhances signals from fluorescent voltage indicators. *Biophys. J.* **120**(23), 5333–5342 (2021). 34710379 10.1016/j.bpj.2021.10.028 PMC8715190

[60] Milosevic, M. M., Jang, J., McKimm, E. J., Zhu, M. H. & Antic, S. D. In vitro testing of voltage indicators: Archon1, ArcLightD, ASAP1, ASAP2s, ASAP3b, Bongwoori-Pos6, BeRST1, FlicR1, and Chi-VSFP-butterfly. *eNeuro*10.1523/ENEURO.0060-20.2020 (2020). 32817120 10.1523/ENEURO.0060-20.2020 PMC7540930

[61] Gonzalez Sabater, V., Rigby, M. & Burrone, J. Voltage-gated potassium channels ensure action potential shape fidelity in distal axons. *J. Neurosci.* **41**(25), 5372–5385 (2021). 34001627 10.1523/JNEUROSCI.2765-20.2021 PMC8221596

[62] Zhou, W. L., Yan, P., Wuskell, J. P., Loew, L. M. & Antic, S. D. Dynamics of action potential backpropagation in basal dendrites of prefrontal cortical pyramidal neurons. *Eur. J. Neurosci.* **27**(4), 1–14 (2008). 18279369 10.1111/j.1460-9568.2008.06075.x PMC2715167

[63] Suresh, J. *et al.* Network burst activity in hippocampal neuronal cultures: the role of synaptic and intrinsic currents. *J. Neurophysiol.* **115**(6), 3073–3089 (2016). 26984425 10.1152/jn.00995.2015 PMC4946605

[64] Meyer-Baese, L., Watters, H. & Keilholz, S. Spatiotemporal patterns of spontaneous brain activity: A mini-review. *Neurophotonics* **9**(3), 032209 (2022). 35434180 10.1117/1.NPh.9.3.032209 PMC9005199

[65] Kerlin, A. *et al.* Functional clustering of dendritic activity during decision-making. *elife*10.7554/eLife.46966 (2019). 31663507 10.7554/eLife.46966 PMC6821494

[66] Yuste, R. & Denk, W. Dendritic spines as basic functional units of neuronal integration. *Nature* **375**(6533), 682–684 (1995). 7791901 10.1038/375682a0

[67] Mainen, Z. F., Malinow, R. & Svoboda, K. Synaptic calcium transients in single spines indicate that NMDA receptors are not saturated. *Nature* **399**(6732), 151–155 (1999). 10335844 10.1038/20187

[68] Ikegaya, Y., Le Bon-Jego, M. & Yuste, R. Large-scale imaging of cortical network activity with calcium indicators. *Neurosci. Res.* **52**(2), 132–138 (2005). 15893573 10.1016/j.neures.2005.02.004

[69] Bandyopadhyay, S., Shamma, S. A. & Kanold, P. O. Dichotomy of functional organization in the mouse auditory cortex. *Nat. Neurosci.* **13**(3), 361–368 (2010). 20118924 10.1038/nn.2490 PMC2866453

[70] Stocca, G., Schmidt-Hieber, C. & Bischofberger, J. Differential dendritic Ca2+ signalling in young and mature hippocampal granule cells. *J. Physiol.* **586**(16), 3795–3811 (2008). 18591186 10.1113/jphysiol.2008.155739 PMC2538933

[71] Kannan, M. *et al.* Fast, in vivo voltage imaging using a red fluorescent indicator. *Nat. Methods* **15**(12), 1108–1116 (2018). 30420685 10.1038/s41592-018-0188-7 PMC6516062

[72] Antic, S. D., Empson, R. M. & Knopfel, T. Voltage imaging to understand connections and functions of neuronal circuits. *J. Neurophysiol.* **116**(1), 135–152 (2016). 27075539 10.1152/jn.00226.2016 PMC4961759

[73] Li, R. *et al.* Holistic bursting cells store long-term memory in auditory cortex. *Nat. Commun.* **14**(1), 8090 (2023). 38062015 10.1038/s41467-023-43620-5 PMC10703882

[74] Bittner, K. C. *et al.* Conjunctive input processing drives feature selectivity in hippocampal CA1 neurons. *Nat. Neurosci.* **18**(8), 1133–1142 (2015). 26167906 10.1038/nn.4062 PMC4888374

