# Supplementary material for: Plateau depolarizations in spontaneously active neurons detected by calcium or voltage imaging
Source: Sci Rep. 2024 Oct 4;14:22787. doi: 10.1038/s41598-024-70319-4 (PMC11452489; doi:10.1038/s41598-024-70319-4)
Supplement: Supplementary file 1 — Supplementary Figures. [file 41598_2024_70319_MOESM1_ESM.pdf]

SUPPLEMENTAL FIGURES

Milicevic et al., 2024

Suppl. Fig. S1

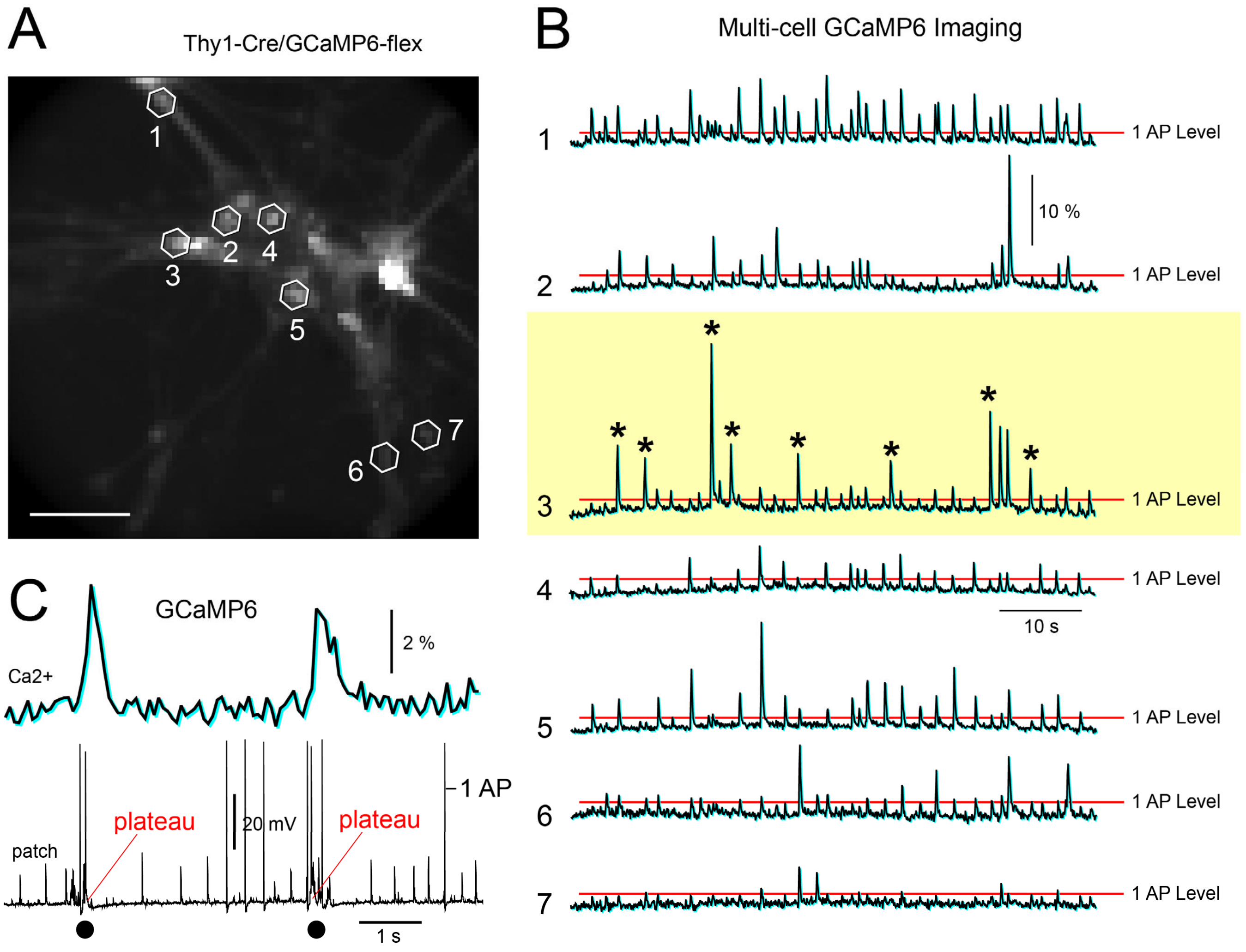

Suppl. Fig. S1. Plateau potentials induce optical Ca2+ transients. (**A**) A cell culture, prepared using Thy1-Cre mice, was transduced with GCaMP6f-flex. Scale bar: 100 µm. (**B**) Optical imaging of spontaneous neuronal activity (at a rate of 14 Hz) was conducted in seven regions of interest (ROIs) simultaneously. The resulting optical signals exhibited highly variable amplitudes over time. We hypothesize that small Ca2+ transients correspond to events involving only a single action potential (AP), while larger optical transients exceeding the "1 AP Level" likely stem from doublets, triplets, and other bursts of action potentials. Very prominent optical signals (indicated by asterisks) are likely attributable to APs occurring in conjunction with plateau depolarizations. (**C**) Dual optical (Ca2+) and electrical (patch) recording was performed from a neuron expressing GCaMP6f. Due to the limited sensitivity of the optical recording, optical Ca2+ transients align well with plateau bursts but do not correspond closely with simple APs lacking a depolarization foot.

++

Suppl. Fig. S2

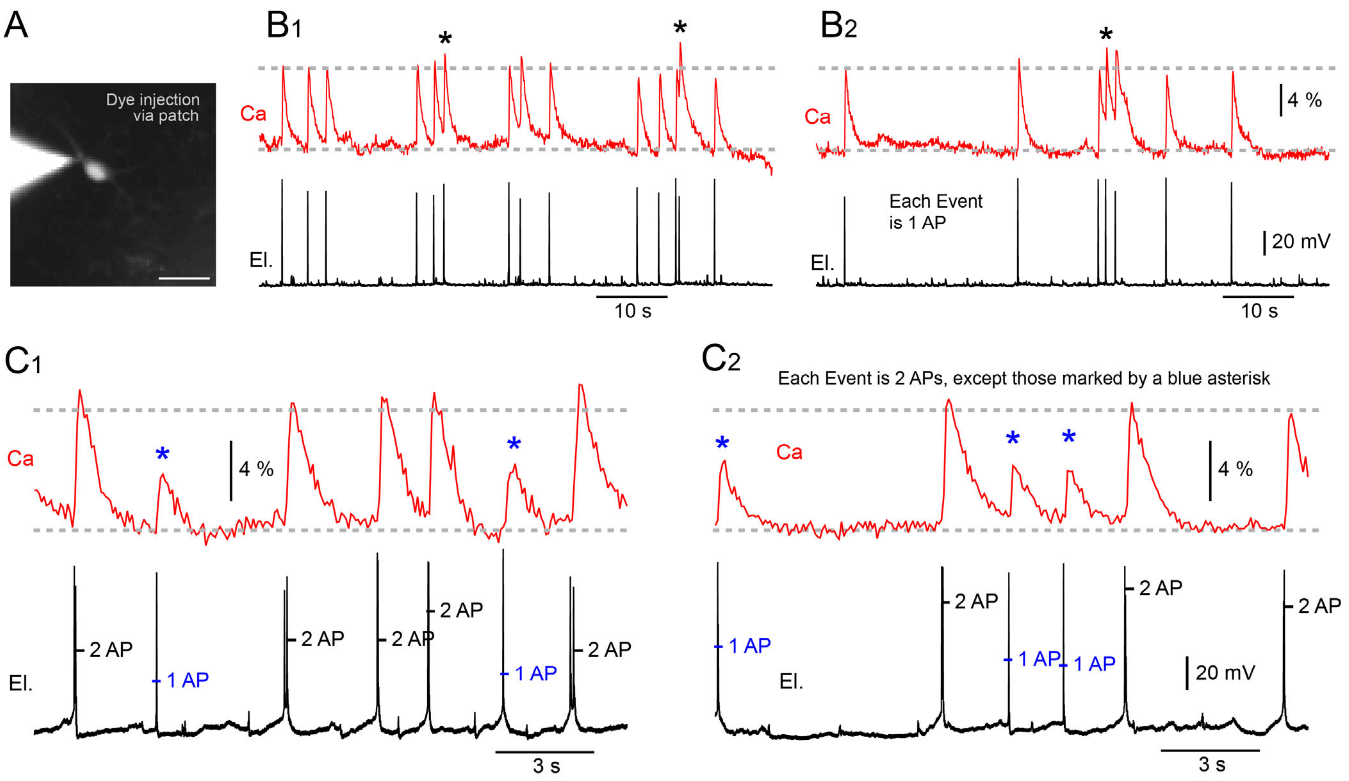

__**Suppl. Fig. S2. Precise relation between electrical and optical signals.**__ (**A**) Only one neuron was loaded with a membrane-impermeable Ca2+-sensitive dye (OGB1, Oregon Green 488 BAPTA-1, hexapotassium salt) via a patch pipette. (**B1**) Dual recordings of spontaneous activity were conducted, with optical signals represented in red and electrical signals in black. Black asterisks indicate transients exhibiting signs of "residual calcium". (**B2**) Same cell and setup as in B1, but the recording was performed 30 seconds later as a consecutive trial. Each electrical event in both B1 and B2 corresponds to a single action potential (1 AP). (**C1**) Similar to B1, but involving a different cell on a different coverslip. Electrical events consist of either a single action potential (1 AP) or two action potentials (2 APs). A blue asterisk marks Ca2+ transients with smaller amplitudes, which coincide with the "1 AP" events. (**C2**) Same cell and setup as in C1, but the recording was conducted 30 seconds later as a consecutive trial.

++

Suppl. Fig. S3

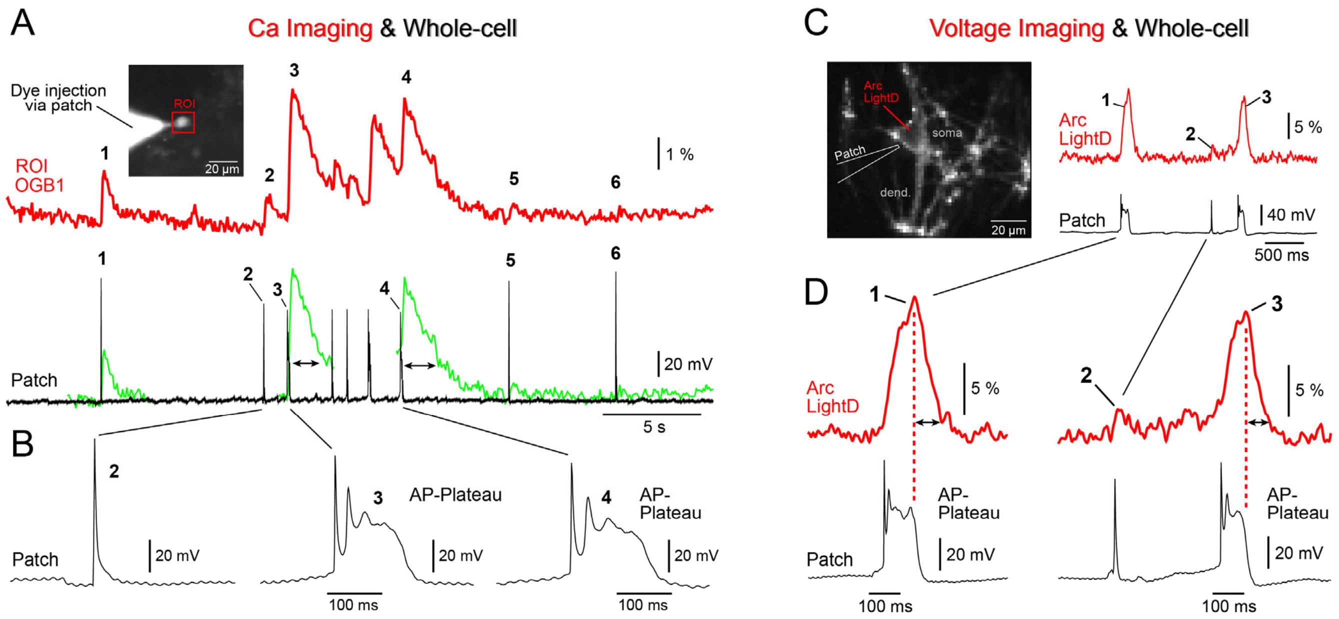

Suppl. Fig. S3. AP-Plateau is a potent inducer of optical signals in both Ca^{2+} and voltage imaging modes. (**A**) A neuron was injected with a Ca2+-sensitive dye (OGB1, 100 µM) via a patch pipette. Dual recordings of spontaneous activity were conducted, with optical signals represented in red (at a rate of 14 Hz) and electrical signals in black. The optical trace was then duplicated, color-coded green, and superimposed onto the electrical trace (black). Double-headed arrows indicate the time period during which the neuronal membrane undergoes complete repolarization, while the Ca2+ optical transient gradually decays towards zero. (**B**) Three electrical events, labeled #2, #3, and #4, are depicted on a finer scale. It's notable that electrical events #2, #5, and #6 consist of single action potentials (1 AP), characterized by weak or brief foot depolarizations. (**C**) A neuron expressing ArcLightD was recorded in two channels: voltage imaging (red, at a rate of 500 Hz) and whole-cell (black trace). (**D**) Three electrical events, labeled #1, #2, and #3, are depicted on a finer scale (patch), along with the corresponding ArcLightD signal. The red dashed vertical line indicates the beginning of the time period during which the neuronal membrane is fully depolarized, while the ArcLightD optical signal slowly decays.

++

Suppl. Fig. S4

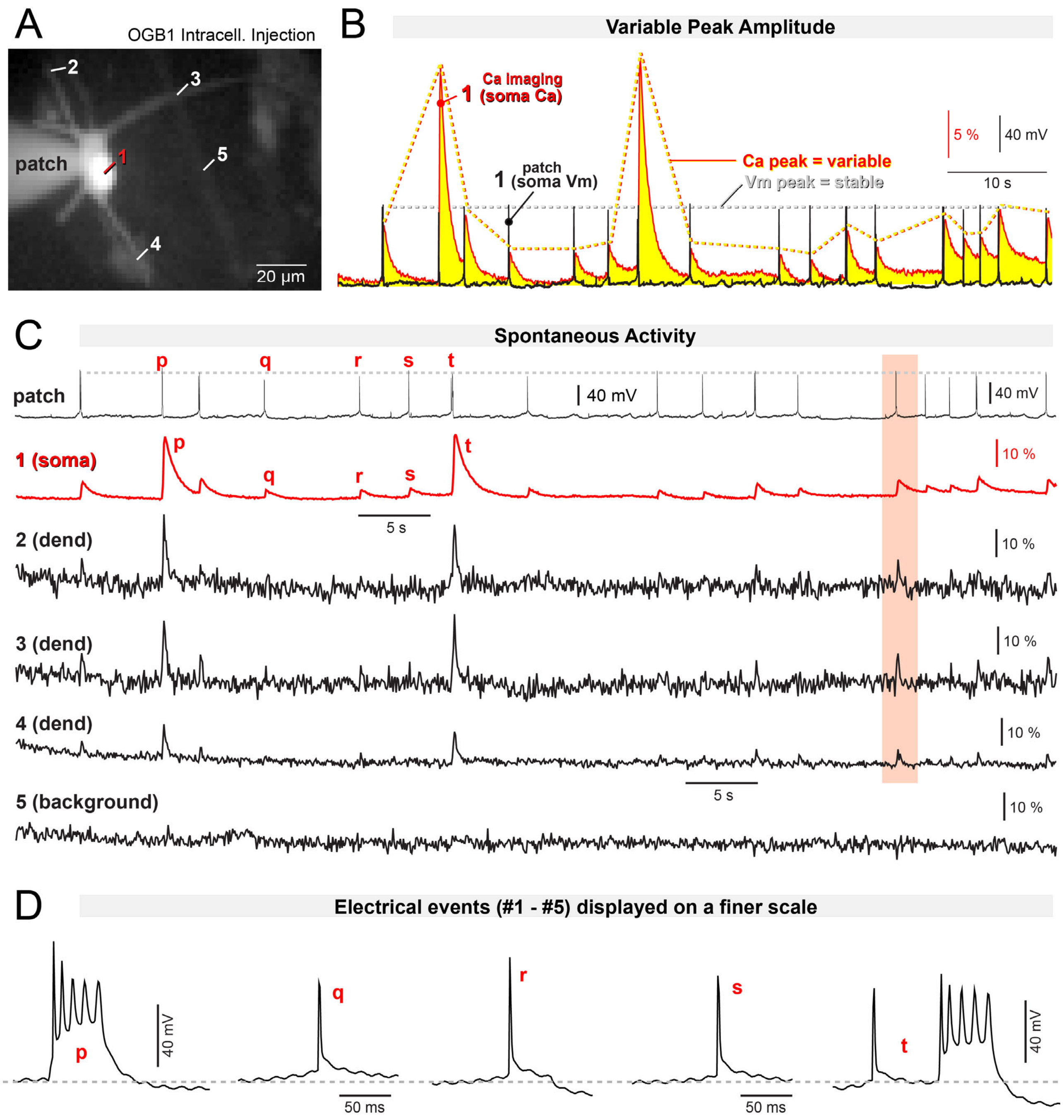

Suppl. Fig. S4. Amplitude variability in Ca2+ imaging experiments. (**A**) A single neuron was loaded with a Ca2+-sensitive dye (OGB1) via a patch pipette. (**B**) Dual recordings of spontaneous activity were conducted, employing both optical (red-yellow) and electrical (black) channels. Each physiological event is simultaneously represented in both recording channels, with notable variability in amplitudes. A dashed gray line connects the peaks of electrical transients in the cell body, emphasizing the uniformity of electrical signals. Additionally, a dashed red-yellow line connects the peaks of subsequent Ca2+ transients in the cell body, highlighting the variability in amplitudes of the corresponding Ca2+ optical signals. (**C**) The same data as presented in *B*, with the addition of Ca2+ transients from dendrites (ROIs #2-4) to the display. A vertical pink stripe draws attention to an electrical event that elicited dendritic Ca2+ transients. ROI #5 samples optical signals in an area devoid of neurites from the patched neuron (background). (**D**) Five electrical events (labeled as "p" to "t") are depicted on a finer scale. Notably, events "p" and "t" represent complex Plateau-Bursts, accompanied by robust Ca2+ transients, as illustrated in panel *C*.

+++

Suppl. Fig. S5

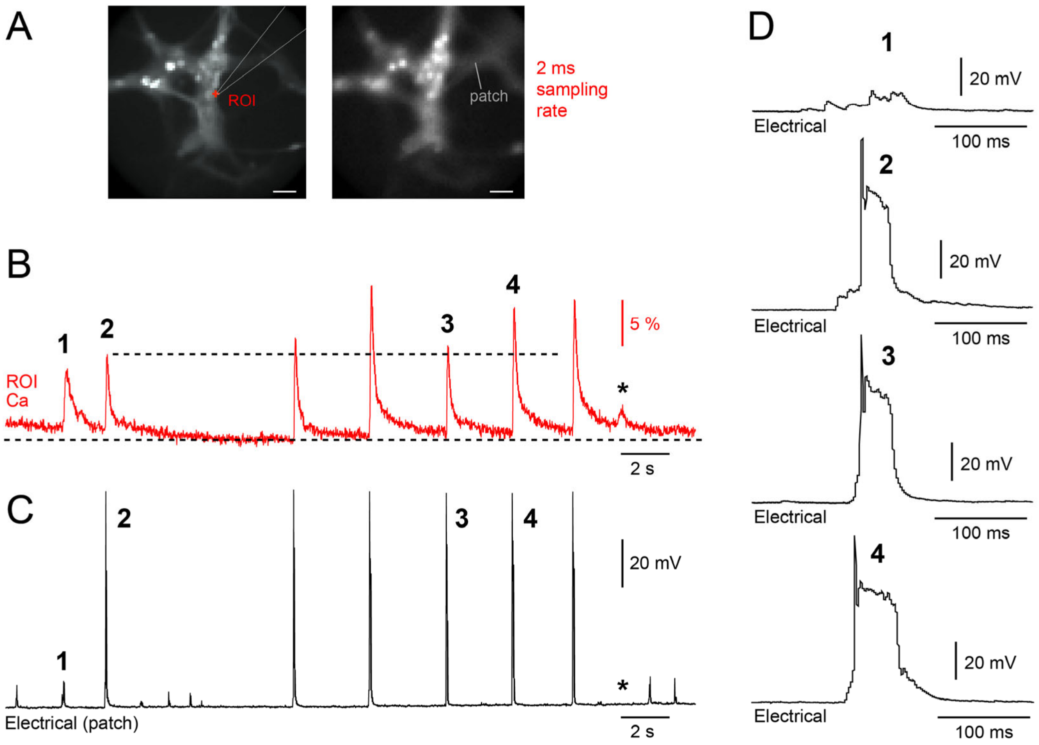

<u>Suppl. Fig. S5. Calcium spikes affect the amplitudes of the Ca2+ transients.</u> (**A**) Left: A single frame extracted from a sequence of 1,500 frames captured at a 2 ms sampling interval (corresponding to a sampling rate of 500 Hz) depicts bulk-loaded neurons in culture, stained with OGB1-AM. Right: The focus is adjusted to reveal the position of the patch pipette filled with a dye-free intracellular solution. Scale bar: 50 μm. (**B**) Ca2+ transients originating from a region of interest (ROI) composed of 5 spatially-averaged pixels, highlighted in red in panel A. (**C**) Whole-cell recording obtained from the cell indicated by "ROI" in panel A. Matching electrical events are numbered consistently in panels B and C. An asterisk denotes a Ca2+ transient that lacks representation in the electrical trace. (**D**) Electrical events #1 - #4 are displayed on a finer scale. Notably, electrical event #4 exhibits a longer duration (half-width) compared to electrical event #3, resulting in a notably greater optical signal amplitude.

++

Suppl. Fig. S6

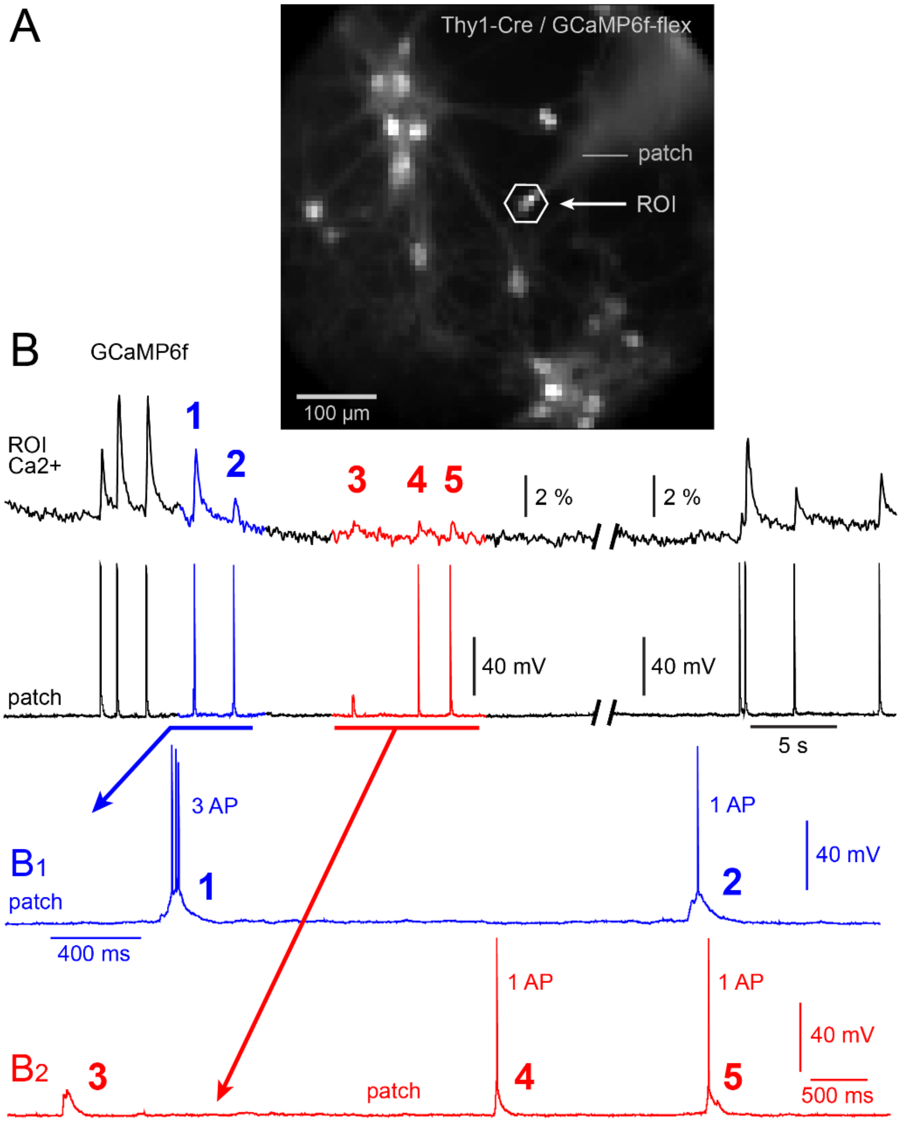

Fig. S6. Sustained depolarizations (plateaus) influence GCaMP6f optical signals. (**A**) A neuron derived from a Thy1-Cre mouse is transduced with GCaMP6f-Flex and patched using a glass pipette (patch). (**B**) Simultaneous dual recordings of spontaneous activity are conducted in the same cell, capturing both optical (Ca2+) and electrical (patch) signals. Five electrical events are selected and labeled accordingly. (**B1**) Events 1 and 2 are depicted on a finer scale. (**B2**) Events 3 through 5 are shown on a finer scale as well. It's noteworthy that weak Ca2+ optical signals #4 and #5 are triggered by single action potentials (1 AP) accompanied by a weak or brief "foot depolarization." Additionally, despite lacking an action potential, subthreshold event #3 has elicited a Ca2+ optical signal.

++

Suppl. Fig. S7

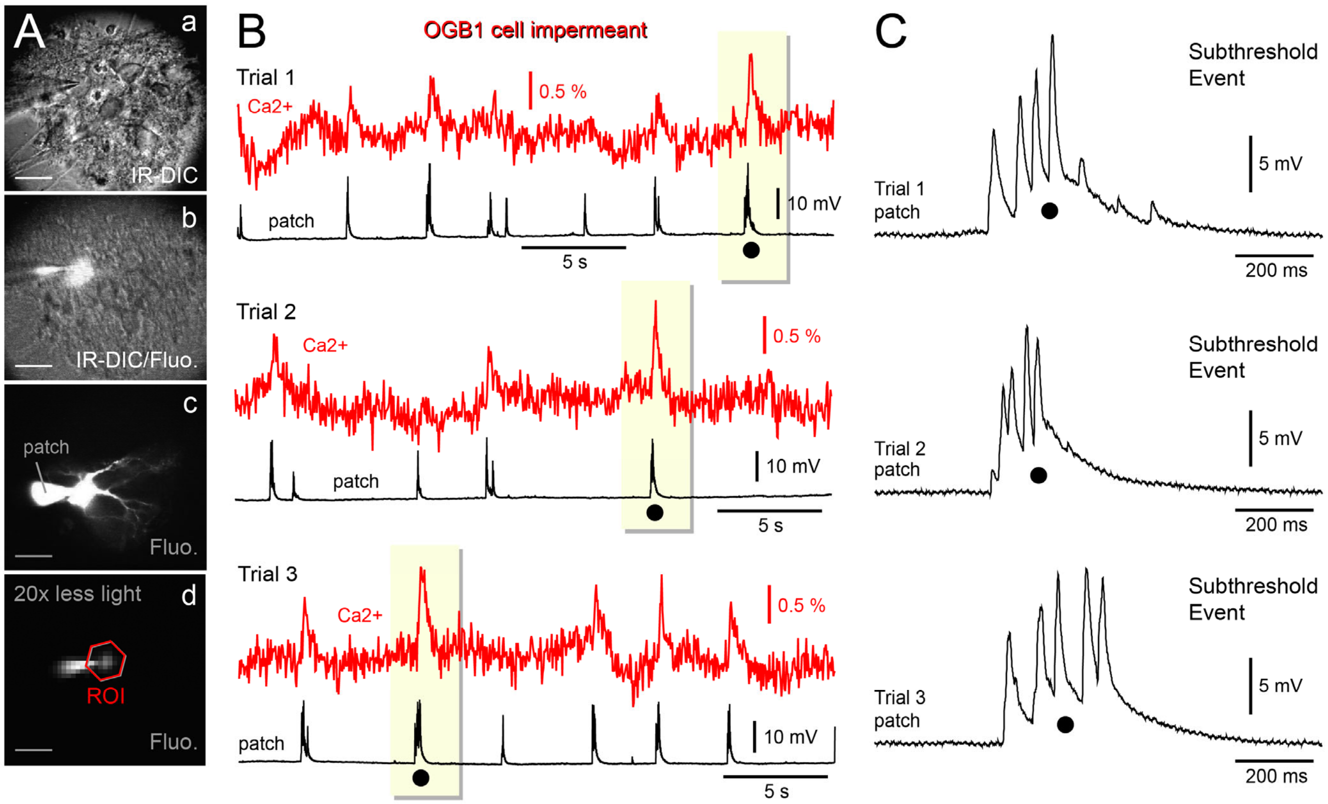

Fig. S7. Even small subthreshold potentials produce optical signals in the Ca^{2+} imaging channel. (**A**) A cultured neuron (Aa) is patched with a glass pipette containing a cell-impermeant form of the Ca2+-sensitive dye, OGB1 (Ab,c). To mitigate phototoxicity, the excitation light during Ca2+ imaging was reduced by a factor of 20 using a neutral density filter (0.05). The neuron's image was projected onto a fast 80x80 pixel camera (Ad) and sampled at a full frame rate of 28 Hz. The region of interest (ROI), containing 15 pixels, is centered on the cell body. (**B**) Dual recordings, representing Ca2+ signals (in red) and whole-cell recordings (in black), captured spontaneous neuronal activity across three consecutive trials (Trials 1-3). The majority of subthreshold events (with amplitudes ranging from 10 to 20 mV) generated optical transients. (**C**) Electrical transients corresponding to conspicuous optical transients are depicted on a finer scale. The electrical events that elicited Ca2+ signals were consistently characterized by a depolarization foot (plateau), indicated by black dots.

++

Suppl. Fig. S8

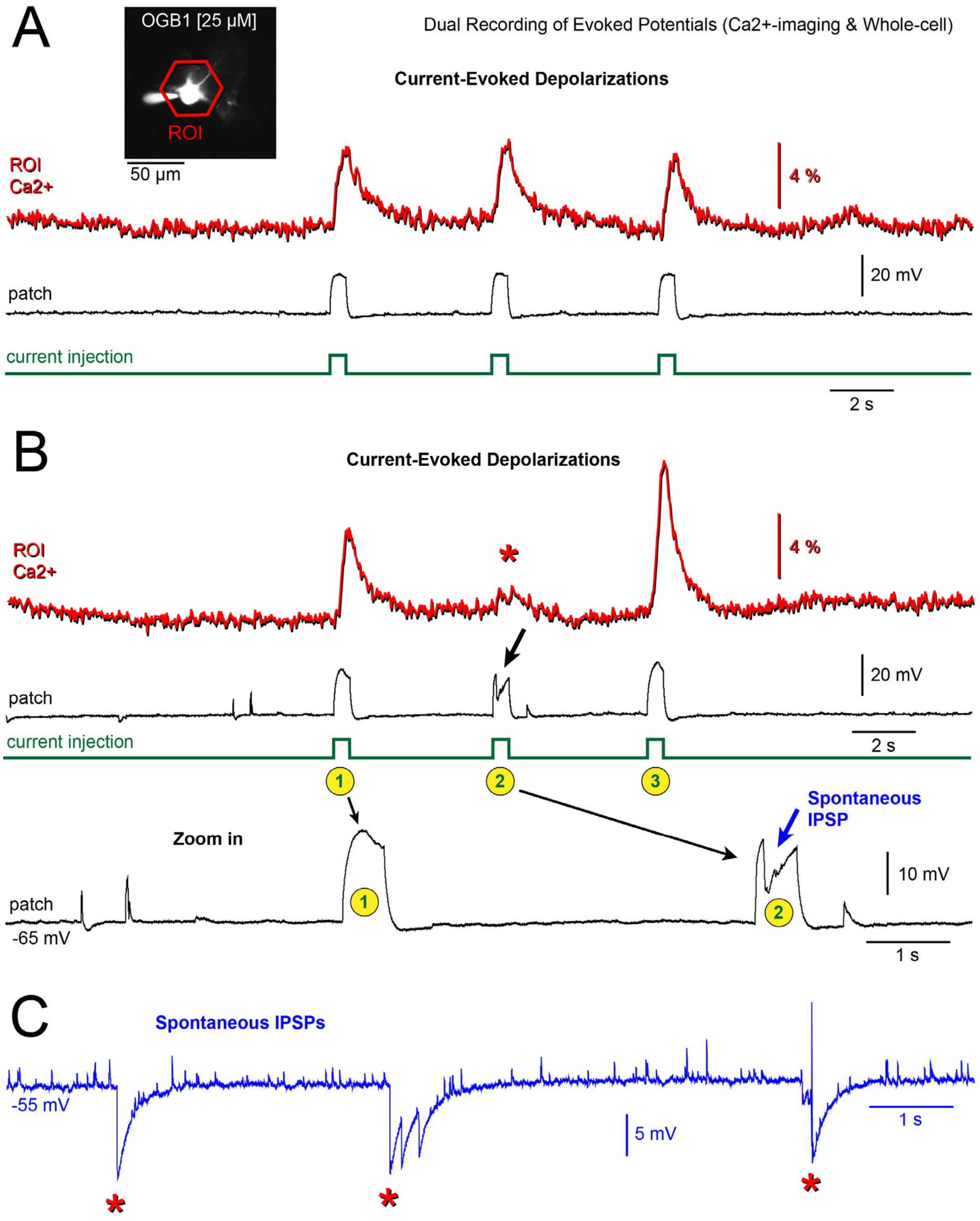

<u>Fig. S8. Subthreshold potentials produce optical signals in the Ca^{2+} imaging channel</u>. (**A**) Dual optical-electrical recording was conducted in a cultured neuron injected with a Ca2+-sensitive dye, OGB1 (25 µM). Three direct current injections into the cell body are represented by the green trace, resulting in neuronal depolarizations (depicted by the black trace) and subsequent Ca2+ transients (illustrated by the red trace). (**B**) Similar to panel A, except an accidental inhibitory postsynaptic potential (IPSP) coincided with the second current injection (#2), leading to a reduction in the Ca2+ transient associated with pulse #2 (marked by a red asterisk). Additionally, a whole-cell recording is provided on a finer scale to highlight the shape of the interfering IPSP (labeled as "Spontaneous IPSP"). (**C**) A whole-cell recording of spontaneous IPSPs is depicted (indicated by red asterisks). The resting membrane potential is displayed beneath the trace for reference.

++

Suppl. Fig. S9

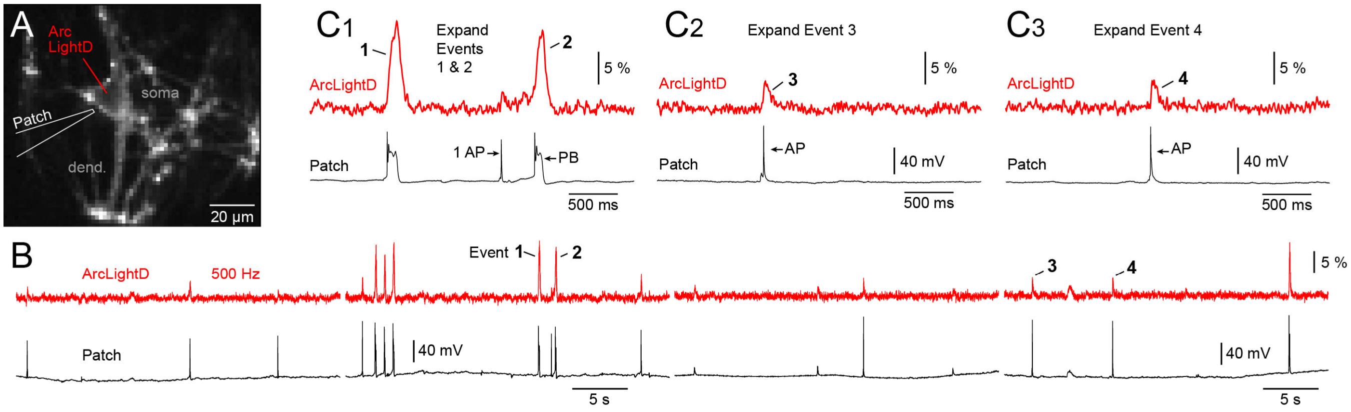

<u>Fig. S9. ArcLightD responds to plateau potentials</u>. (**A**) A cortical neuron expressing ArcLightD was patched and projected onto a fast CCD camera using a 40x objective. Unprovoked spontaneous electrical activity was recorded in two channels: [i] optically using ArcLightD voltage imaging (red trace, 500 Hz) and [ii] electrically via a whole-cell configuration (patch). (**B**) The optical (red trace) and electrical (black trace) channels are displayed over a 120-second period (comprising 4 sweeps of 30 seconds each). (**C1**) Events #1 and #2 are presented on a finer scale. Notice the amplitude difference between a single action potential (1 AP) and an action potential combined with a plateau depolarization (Plateau-Burst). (**C2-3**) Events #3 and #4 are shown in detail. Observe that single action potentials produce small optical signals.

++
